# Charging effect induced by electron beam irradiation: a review

**DOI:** 10.1080/14686996.2021.1976597

**Published:** 2021-11-11

**Authors:** Z.J. Ding, Chao Li, Bo Da, Jiangwei Liu

**Affiliations:** a Department of Physics and Hefei National Laboratory for Physical Sciences at Microscale, University of Science and Technology of China, Hefei, People’s Republic of China; b Research and Services Division of Materials Data and Integrated System, National Institute for Materials Science, Tsukuba, Japan; c Research Center for Advanced Measurement and Characterization, National Institute for Materials Science, Tsukuba, Japan; d Research Center for Functional Materials, National Institute for Materials Science, Tsukuba, Japan

**Keywords:** Charging effect, Monte Carlo, experiment, electron beam irradiation, 70 New topics/Others, 202 dielectrics / piezoelectrics / insulators, 212 surface and interfaces, 502 electron spectroscopy

## Abstract

Charging effect frequently occurs when characterizing nonconductive materials using electrons as probes and/or signals and can impede the acquisition of useful information about the material under investigation. It is not adequate to investigate it merely by experiments, but theoretical investigations, for which the Monte Carlo method is a suitable tool, are also necessary. In this paper we review Monte Carlo simulations and selected experiments, intending to provide general insight into the charging effects induced by electron beam irradiation. We will introduce categories of the charging effect, the theoretical framework that is adopted in Monte Carlo modeling of the charging effect and present some typical simulation results. At last, with the knowledge on charging effect imparted by the above contents, we will discuss the measures that can be used for minimizing it.

## Graphical Abstract

**Figure:**
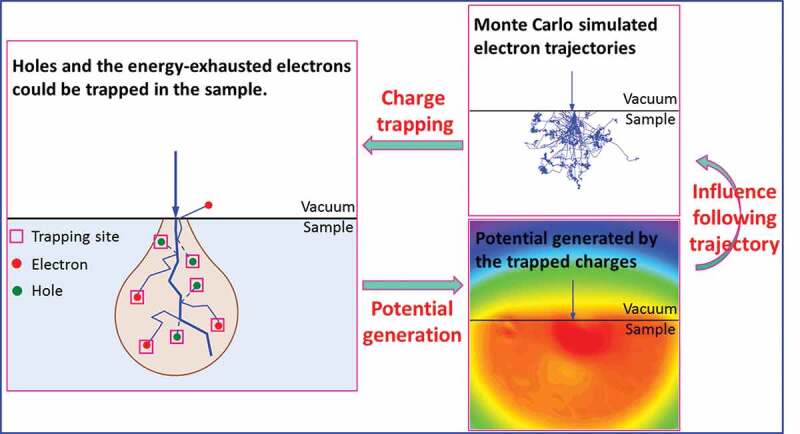

## 1. Introduction

Most kinds of condensed matter in this planet are nonconductive materials; they are, for example, minerals, ceramics, halides, oxides, glasses, polymers and biomaterials in different forms of bulk solid, porous solid or foam, particle, fiber, film, liquid and soft matter. Some insulating materials have been playing important roles in high-tech fields or modern industry owing to their special mechanical, electrical, chemical, electronic, optical and thermal properties. One example is SiO_2_ as the gate oxide layer and the insulation layer between adjacent conductive parts in manufacturing integrated circuits. And by electron beam lithography (EBL) technique for manufacturing microstructures, different kinds of insulating materials are used for the indispensable resist layer [1–3]. Besides, insulating materials are important for the development of new devices, e.g. energy storage capacitors [4,5] and gas sensors [6,7]. For the insulators a remarkable behavior under irradiation of different types of energetic particles (electrons, neutrons, ions and photons) is the electrostatic charging phenomenon, which has attracted considerable research interest in the development of controlled nuclear fusion reactor [8] and for satellites and spacecrafts protection [9]. Among them the electron irradiation-induced charging effect is particularly important for surface analysis [10,11] and microbeam analysis [12,13].

Functions of the manufactured devices are closely related to their size, geometry, and composition, highlighting the importance of characterizing them by using the electron beam based techniques, such as, scanning electron microscopy (SEM) and Auger electron spectroscopy (AES). Since the principle of these characterization techniques is based on electron-solid interaction, it is crucial to comprehensively investigate the interaction of the electron beam with insulating materials both experimentally and theoretically for accurate characterization of manufactured devices containing insulating materials.

However, unusual phenomena have been observed when irradiating insulating materials with an electron beam for investigating the interaction. For example, the measured electron yields of different insulating materials show a time-dependent characteristic: as the irradiation goes on the electron yield decreases if the beam energy is low [14–16], and increases if the beam energy is high [14]. As for the curve of electron yield versus the beam energy, it may deviate from the conventional shape to a large extent [16,17] due to such a time-dependent characteristic. When using SEM for characterizing insulating materials some problems may also occur: in addition to the distortions [18–22] and the anomalous white/dark contrast [18,22–27] shown in the secondary electron images, the contrast can vary a lot with the beam energy [28] and even magnification. Other unusual phenomena include EBL pattern distortion [29–31] and shift of the secondary electron peak in the energy spectrum [28,32]. In fact, all these unusual phenomena are manifestations of the charging effect, which, in principal, is due directly to the accumulation of an amount of static electric charges in the insulating sample. Such a charge accumulation may maintain for a long time due to poor conductivity of the sample, and can generate an electric field inside and outside the sample, affecting the incidence of primary electrons, the detection of secondary and backscattered electrons, as well as the transport of electrons inside the sample. Though in some special cases the sample structure can be better known from their secondary electron images with occurrence of the charging effect [33–35], it is still undesired in most cases because it impedes the acquisition of useful information of the sample under characterization. It is thus necessary to investigate in detail the physical cause and the quantitative characteristics of the charging effect, which can help researchers to assess its influence on associated experiments and to develop measures to minimize it.

For its physical cause, it has been known that charge trapping plays a fundamental role in the formation of the charging effect [36–41]. Specifically, in a solid sample, defects, impurities, vacancies, dislocations, or any regions with inhomogeneous dielectric constants [42] can be regarded as the trapping sites, which have the capability of trapping electrons and holes. The volume density of the trapping sites, however, depends strongly on the crystallinity: it is in the order of 10^16^ cm^−3^ for crystalline materials, 10^17^–10^20^ cm^−3^ for polycrystalline materials, and 10^22^ cm^−3^ for amorphous materials [41]. On the other hand, the trapping sites can be identified to be Coulombically attractive, repulsive, or neutral [43]; for electrons, a Coulombically attractive trapping site is the one occupied by a positive charge, a Coulombically repulsive trapping site is the one occupied by a negative charge, and a neutral trapping site is the one without any charge in it. Each kind of the trapping site can be characterized by its trapping cross section and trapping energy. For the trapping cross-section, the Coulombically attractive trapping site has the greatest magnitude, ~10^−12^–10^−14^ cm^2^; the neutral trapping site has the moderate one, ~10^−14^-10^−18^ cm^2^; the Coulombically repulsive trapping site has the lowest one, ~10^−18^-10^−21^ cm^2^ [43]. In particular, the trapping cross-section can be measured by experiments, say, by using the low-energy electron transmission (LEET) technique, Bass et al. [44,45] have measured the electron trapping cross section of the material which is with a sub-monolayer quantity and is deposited on a multi-layer insulating film. Basically, the multi-layer film is supported by a metallic substrate, so in using an electron beam with an adjustable low energy to irradiate it, a sharp increase in the transmission current will be detected in LEET, known as the injection current (IC), when the incident electron energy exceeds a threshold to enable the incident electron to penetrate the insulating film. However, the electron trapping in the deposited sub-monolayer material, produced prior to the IC measurement by using an electron beam with a definite low energy to irradiate it, induces a negative surface potential and will shift the IC position to the higher energy side. The shift magnitude of the IC position can be obtained in LEET and naturally carries the information of the electron trapping cross section. For a particular kind of the trapping site, however, its trapping cross section can be varied by an electric field, say, Ning [46] has found experimentally that in SiO_2_ the trapping cross section of the Coulombically attractive trapping site for electrons can be reduced by as much as two orders of magnitude when the imposed electric field is increased from 0.5 to 3 MV cm^−1^, owing to the electron heating and the reduction of the trapping energy. Besides, Tzou et al. [47] have found that the trapping cross-section of the neutral trapping site for holes can also be reduced by imposing an electric field on the SiO_2_ sample. On the other hand, the energy level of a trapping site should be within the forbidden band of the insulating material and the trapping energy, i.e. the energy difference between the trapping level and the conduction band bottom, has a typical range of 0–3 eV [48–50]. However, a trapped electron or hole can be released from the trapping site if it can gain from any source an energy exceeding the trapping energy.

The electron beam energy used in experiments is often much greater than the trapping energy, say, the primary electron energy in SEM is usually in the range of ~0.1–30 keV. Definitely, the trapping sites are not able to trap the primary electrons just incident into the sample, but charge trapping does occur, implying the electron transport inside the solid sample plays also an important role in the formation of the charging effect and therefore the knowledge regarding it is necessary. It has been known that the electrons transporting in a solid sample interact not only with the atomic nucleus and electrons, but also with the lattice vibrations, i.e. phonons. Electron-nucleus interactions deflect electron trajectories, electron-electron interactions excite other signals, such as secondary electrons, Auger electrons, as well as X-rays, and electron-lattice interactions create or annihilate phonons. In particular, the energy of the transporting electrons will be partially lost due to electron-electron and electron-lattice interactions, such that it will polarize the region surrounding it when its energy becomes rather low, leading to the increase in its effective mass and the formation of a polaron. In contrast, holes can be regarded as polarons as soon as they are generated in secondary electron excitations, due to their great effective mass. These polarons cannot escape the sample due to their lack of energy and therefore are essentially deposited inside the sample, but they can still drift in an electric field until they get trapped or recombined at the trapping sites, finally giving rise to a charge accumulation by which the electric field is generated.

The above discussions impart qualitative knowledge on the formation of the charging effect, whereas quantitative knowledge still lacks. It is, however, almost impossible to reveal the comprehensively quantitative characteristics of the charging effect exclusively by experiments, due to the difficulty concerned with measuring the important physical quantities, such as, the distribution of the potential and the electric field inside the sample. Thus, it has to resort to theoretical tools to compensate the deficiency of experimental investigations, and so far different theoretical models have been available. For example, Melchinger and Hofmann [51] have proposed a dynamic double-layer model (DDM) to describe the charge distribution induced by electron beam irradiation, which assumes positive charges to develop in an axisymmetric cylindrical volume at the surface and negative charges to accumulate at a certain depth below the sample surface. This model, however, is essentially one-dimensional one because it not only requires the electron beam to be defocused but also requires the sample surface to be flat. Refs. [52–56] refer to another one-dimensional model that describes the related physical processes, such as, primary electron beam injection, secondary electron emission (SEE), charge transport and charge trapping, etc., as electric currents flowing perpendicular to the sample surface. Naturally, the applicability of those one-dimensional models is not very broad since appreciable samples characterized in experiments have complex structures. In contrast, a Monte Carlo method can trace the transport of the electrons incident into a sample with a complex structure or geometry. And it allows the incorporation of the excitation of various signals, i.e. secondary electrons [57–62], Auger electrons [63–66], and X-rays [67,68] into the simulation. Furthermore, the Monte Carlo method has been proven reliable in investigating electron transport or electron–solid interactions even when the electron energy is as low as several eV [69] or as high as hundreds of keV [70]. And it is not physically difficult to incorporate the charge trapping into the simulation, which, in conjunction with the superior capabilities of the Monte Carlo method mentioned above, enables it to be an ideal theoretical tool for investigating the charging effect.

This paper will review the reported investigations on the charging effect, including Monte Carlo simulations and experiments, with the intention of helping readers to gain general insights into the charging effect induced by electron beam irradiation.

## 2. Electron transport theory

It has to be clear that the accurate description of electron transport in a solid sample is crucial for simulating charging effect. In Monte Carlo simulations, the complicated electron transport is simplified into several types of scattering: elastic scattering due to electron-nucleus interaction, inelastic scattering due to electron–electron interaction, and phonon scattering due to electron-lattice interaction. In what follows, we will present the theoretical models describing these scatterings, which are crucial for Monte Carlo simulations. In particular, the transport trajectory of an electron can be obtained via sequentially connecting the positions where the scatterings occur [71,72].

### 2.1. Elastic scattering

**Figure 1.**
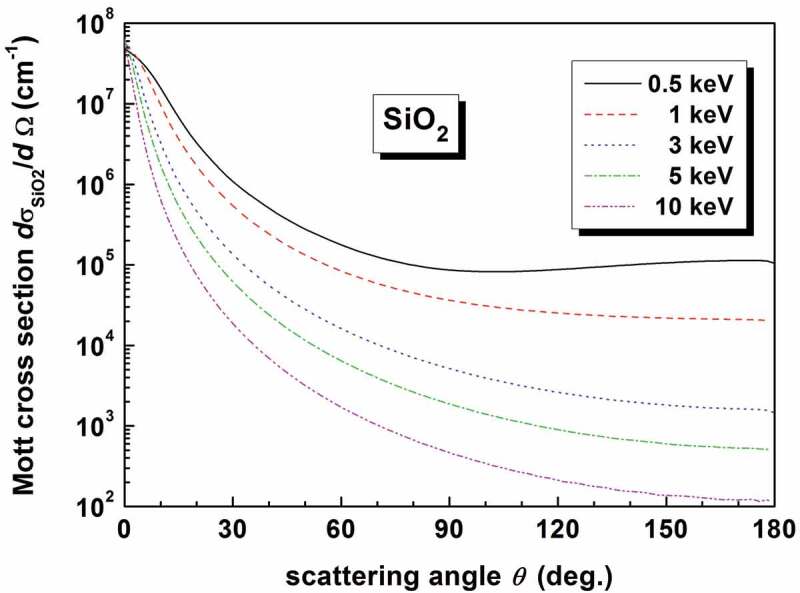
Calculated Mott differential cross-sections for elastic collisions of electrons at different energies of 0.5, 1, 3, 5 and 10 keV in SiO_2._

The Mott’s cross section [73] is known to be the most accurate to describe electron elastic scattering via the solution of Dirac equation. The differential cross-section for an electron scattered into polar angle θ within a solid angle dΩ is given by,

(1) $$\frac{d\sigma_{e}}{d\Omega }={\left|f\left(\theta \right)\right|}^{2}+{\left|g\left(\theta \right)\right|}^{2},$$

where the scattering amplitudes

(2) $$\begin{array}{rl} & f\left(\theta \right)\\ & =\frac{1}{2ik}\sum_{\ell =0}^{\infty }\left\{\left(\ell +1\right)\left(e^{2i\delta_{\ell }^{+}}-1\right)+\ell \left(e^{2i\delta_{\ell }^{-}}-1\right)\right\}P_{\ell }\left(\cos\theta \right);\\ & g\left(\theta \right)=\frac{1}{2ik}\sum_{\ell =1}^{\infty }\left\{-e^{2i\delta_{\ell }^{+}}+e^{2i\delta_{\ell }^{-}}\right\}P_{\ell }^{1}\left(\cos\theta \right), \end{array}$$

can be calculated using the partial wave method [74]. In Equation (2), $P_{\ell }\left(\cos\theta \right)$ and $P_{\ell }^{1}\left(\cos\theta \right)$ are respectively the Legendre function and the first-order associated Legendre function, and $\delta_{\ell }^{+}$ and $\delta_{\ell }^{-}$ are respectively the spin-up and spin-down phase shifts of the ℓth partial wave. The Thomas-Fermi-Dirac atomic potential [75] may be used. To gain some direct insights into the Mott’s cross-section, we present in Figure 1 the differential cross-sections at different energies for SiO_2_ by

(3) $$\begin{array}{c} \frac{d\sigma_{SiO_{2}}}{d\Omega }\\ =\rho_{SiO_{2}}N_{A}\left(\frac{d\sigma_{Si}}{d\Omega }\frac{a_{Si}r_{Si}}{a_{Si}r_{Si}+a_{O}r_{O}}\frac{1}{a_{Si}}+\frac{d\sigma_{O}}{d\Omega }\frac{a_{O}r_{O}}{a_{Si}r_{Si}+a_{O}r_{O}}\frac{1}{a_{O}}\right), \end{array}$$

where $\rho_{SiO_{2}}$ is the density of SiO_2_ and $N_{A}$ is the Avogadro constant; $d\sigma_{Si}/d\Omega$ and $d\sigma_{O}/d\Omega$ are the Mott’s differential cross-sections calculated by Equations (1) and (2) for the collision with a single Si- and O-atom, respectively; a is the relative atomic mass, and $r_{Si}=1/3$ and $r_{O}=2/3$ are the atomic ratios for Si and O elements in SiO_2_. It is seen that it low-energy electrons is more likely to experience large-angle elastic scattering.

An open source software, ELSEPA, developed by Salvat et al. [76] has been available for calculating the Mott’s cross section of electron elastic scattering by using the partial wave method. ELSEPA includes more interaction potentials by taking into account the local exchange interaction, the charge polarization, as well as the depletion of the projectile wave function by open inelastic channels. On the other hand, ELSEPA by default uses the numerical Dirac-Fock electron density for neutral free atoms, which is the most accurate electron density available. It also allows choosing different approximate analytical electron densities for neutral atoms, including the Thomas-Fermi-Molière density, the Thomas-Fermi-Dirac density, and the Dirac-Hartree-Fock-Slater density.

### 2.2. Inelastic scattering

The dielectric functional approach can be used to model individual electron inelastic scattering event by including all inelastic channels in an identical formulation, with which the double differential cross-section is expressed as,

(4) d2λin−1dℏωdq=1πa0EIm−1εq,ω1q,

where $\lambda_{in}$ is the electron inelastic mean free path (IMFP), ℏω the energy loss, ℏq the momentum transfer, $a_{0}$ the Bohr radius, E the electron energy and $\varepsilon \left(q,\omega \right)$ the dielectric function of the sample. The q-dependent energy loss function (ELF), Im−1/εq,ω, can be extended from the optical limit at q=0 modeled with a sum of Lorentz oscillators [77]:

(5) Im−1εq,ω=Im−11+∑jχjq,ω,

where $\chi_{j}\left(q,\omega \right)$ is the electric susceptibility of the *j*th oscillator and can be given by

(6) $$\chi_{j}\left(q,\omega \right)=\Omega_{j}^{2}\frac{f_{j}}{\omega_{j}^{2}\left(q\right)-\omega^{2}-i\omega \Gamma_{j}\left(q\right)},$$

where $\Omega_{j}=\sqrt{4\pi n_{j}e^{2}/m}$ is the plasmon frequency for electron concentration $n_{j}$, e the electron charge and m the electron mass; $f_{j}$, $\omega_{j}\left(q\right)$ and $\Gamma_{j}\left(q\right)$ are the oscillator parameters, i.e. strength, frequency and lifetime, respectively. The optical ELF, Im−1/ε0,ω, is fitted to experimental data [78] with Equation (5) to obtain the oscillator parameters at q=0. Then the ELF values at q≠0 can be obtained by extending the optical ELF with the dispersion relations,

(7) $$\hbar \omega_{j}\left(q\right)=\hbar \omega_{j}\left(0\right)+\alpha \hbar^{2}q^{2}/m,$$

and

(8) $$\Gamma_{j}\left(q\right)=\Gamma_{j}\left(0\right)\left(1+\beta q^{2}\right),$$

where α and β are material-dependent constants (α≈0 and β≈6$A^{2}$ for SiO_2_). Other models [79–81] also extrapolate the ELF from *q* = 0 to *q* ≠ 0 either in a way of continuous integration or of finite summation with the expansion basis of Lindhard dielectric function or Mermin dielectric function. In the Penn’s model, the single-pole approximation (SPA) for Lindhard dielectric function is often used to simplify the calculation [72,82,83] by assuming an explicit dispersion relation. While the full Penn’s algorithm (FPA) [60] considers the full expression of Lindhard dielectric function and does not require any dispersion relation. Errors can be introduced when applying the SPA to low-energy region, limiting its application to the energy range >200 eV [79], while the FPA does not have such a limitation. Both the SPA and FPA need the experimental data of optical constants for optical ELF, but neither of them needs the numerical fitting procedure for the optical ELF. However, in other models the optical ELF is fitted by summing over a series of Drude- [81] or Mermin-type ELFs [80], from which the parameters of each Drude- or Mermin-type ELF are obtained. Da et al. [69] have used a large number of Mermin-type ELFs to fit the optical ELF for taking into account the phonon and inner-shell excitations. A dispersion relation is needed for Drude-type ELF but none for Mermin-type. Da et al. [84] have compared the FPA and Mermin models and found that the difference between them is mainly in very low-energy region, because the finite plasmon lifetime is omitted in the FPA model. It is thus clear that the optical data from which the ELF is derived are crucial for the modelling of electron elastic scattering at low energies, and, particularly for secondary electrons. In addition to the measurement with optical methods, the optical data can also be extracted from the reflected electron energy loss spectroscopy (REELS) spectrum by using either an analytical deconvolution formula [85,86] or more accurately by a reverse Monte Carlo (RMC) method [87–93].

To take into account the bandgap of semiconducting and insulating materials, Equation (5) is rewritten as,

(9) Im−1εq,ω=Im−11+∑jχjq,ωΘℏω−Eg,

where the Heaviside step function $\Theta \left(\hbar \omega -E_{g}\right)$ indicates that the energy loss must be greater than the band gap $E_{g}$ [94] such that the electrons in the valence band can be excited to conduction band and, thus, forming secondary electrons.

**Figure 2.**
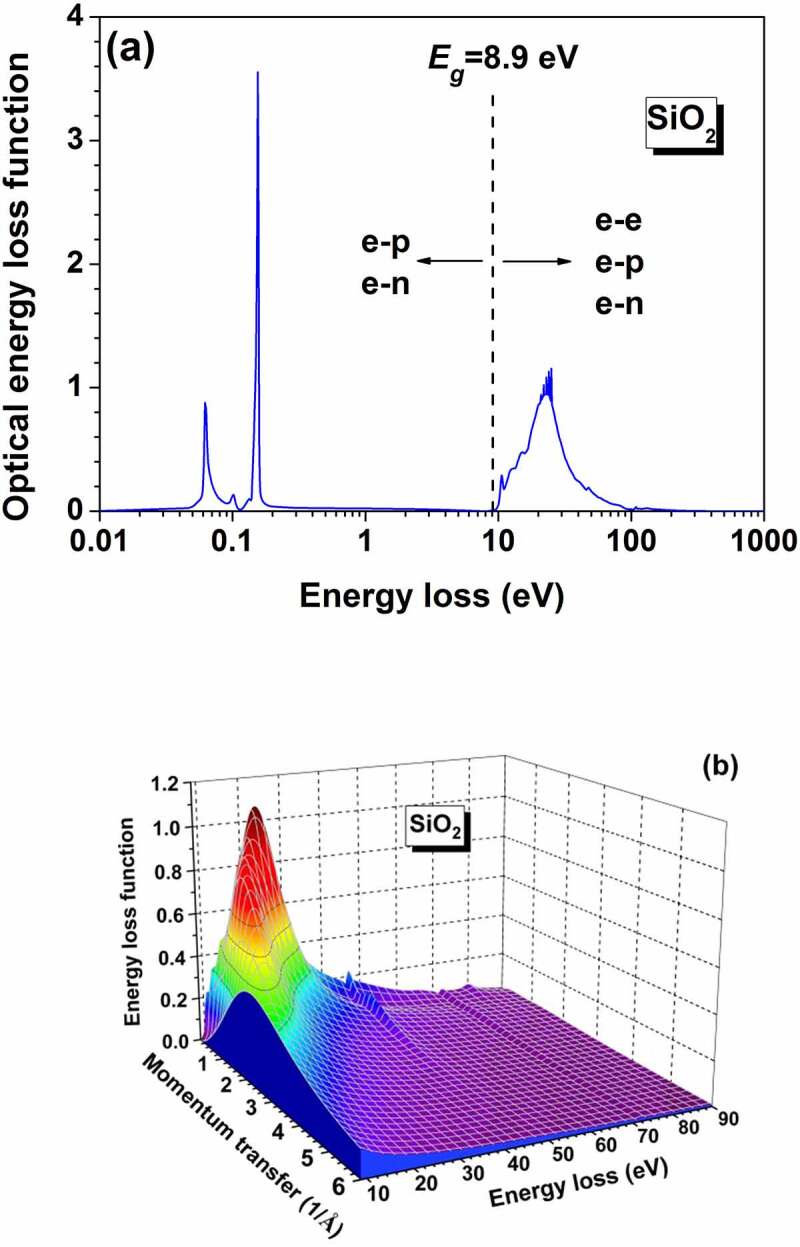
(a) Optical energy loss function of SiO_2_ [78]. The bandgap of SiO_2_ is 8.9 eV, below which the electron-electron interaction is absent. (b) Extrapolated *q*-dependent energy loss function of SiO_2._

The optical ELF and extrapolated *q*-dependent ELF of SiO_2_ are shown respectively in Figure 2(a,b). The ELF in Figure 2(a) is quite small in the range $0.2\;eV<\hbar \omega <E_{g}$ owing to the absence of electron–electron interaction channel. Two sharp energy loss peaks appear below 0.2 eV, which are related to the energy loss for electron–lattice interaction, i.e. the phonon excitation.

Physically, the energy loss of the transport electron in an inelastic scattering event will be transferred to the sample, leading to the excitation of a secondary electron. Applying the FPA model for metals [60], two different mechanisms are considered for the secondary electron excitation, i.e. single-electron excitation and via plasmon damping [95], occurring in the range of $q^{-}\left(\omega ;\omega_{p}\right)<q<q^{+}\left(\omega ;\omega_{p}\right)$ and $q<q^{-}\left(\omega ;\omega_{p}\right)$, respectively, where $q^{-}\left(\omega ;\omega_{p}\right)$ and $q^{+}\left(\omega ;\omega_{p}\right)$ are the left and right boundaries of the non-zero region of the imaginary part of the Lindhard dielectric function, respectively, and $\omega_{p}$ is the plasmon frequency. In the single-electron excitation, the probability for an electron in the Fermi sea with a momentum $\hbar k_{i}$ to be excited to the final state $\hbar k_{f}$ ($k_{f}=k_{i}+q$) is given by

(10) $$\begin{array}{rl} & p\left(\left|k_{i}\right|<k_{F};q,\omega \right)=\int dk_{i}\delta \left[\hbar \omega -\frac{\hbar^{2}}{2m}\left(2k_{i}\cdot q+q^{2}\right)\right],\\ & \;\;\;\;\;\;\;\;\;\;\;\;\;\;\;\times \Theta \left(k_{F}-\left|k_{i}\right|\right)\times \Theta \left(\left|k_{f}\right|-k_{F}\right) \end{array}$$

where ℏq is momentum transfer, ℏω the energy loss, $\hbar k_{F}$ the Fermi momentum. Due to the requirement of momentum and energy conservation, the energy of the Fermi sea electron and the excited secondary electron can be given, respectively, by

(11) $$\begin{array}{rl} & \frac{\hbar^{2}k_{i}^{2}}{2m}=\frac{\hbar^{2}}{2m}\left(k_{x}^{2}+k_{x}^{2}+k_{x}^{2}\right)=E^{\prime };\\ & \frac{\hbar^{2}k_{f}^{2}}{2m}=\frac{\hbar^{2}}{2m}{\left(k_{i}+q\right)}^{2}=E^{\prime }+\hbar \omega . \end{array}$$

As for plasmon damping mechanism, Chung and Everhart proposed that the probability for exciting a Fermi sea electron with an energy $E^{\prime }$ to be a secondary electron with an energy $E^{\prime }+\hbar \omega$ is proportional to the joint density of states, $p\left(E^{\prime },\hbar \omega \right)\propto \sqrt{E^{\prime }\left(E^{\prime }+\hbar \omega \right)}$ [96].

The probability for a secondary electron with an energy *E* to transmit through the surface of a metal is expressed as [72],

(12) $$T\left(E,\beta \right)=\left\{\begin{array}{c} \frac{4\sqrt{1-U_{0}/E\cos^{2}\beta }}{{\left[1+\sqrt{1-U_{0}/E\cos^{2}\beta }\right]}^{2}}\;\;\;\;\left(E\cos^{2}\beta >U_{0}\right);\\ 0\;\;\;\;\;otherwise, \end{array}\right.$$

where $U_{0}=E_{F}+\Phi$ is the inner potential, $E_{F}$ the Fermi energy and Φ the work; β is the angle of the velocity of a moving electron in relative to the surface normal.

The FPA model discussed above has also been applied to calculate the electron yield of semiconducting materials [97], where the energy reference for semi-conducting materials is taken as the bottom of the conduction bands and the surface barrier controlling the SEE is replaced by the electron affinity χ, i.e. $U_{0}=\chi$.

As for insulating materials, the energy of the excited secondary electron can be simply taken as $E=\hbar \omega -E_{g}$, and, the initial transport direction as that of the momentum transfer, which essentially describes the excitation of a bound electron at the valence band top to a free state in the conduction band. This approach has been adopted in Ref [98]., the calculated electron yields of SiO_2_ agree with experiments. Ganachaud et al. [99] have treated the secondary electron excitation in insulating materials with the joint density of states, $p\left(E^{\prime },\hbar \omega \right)\propto n\left(E^{\prime }\right)n\left(E^{\prime }+\hbar \omega \right)$, which describes the probability for an electron in the valence band at $E^{\prime }$ to be excited to a free state at $E^{\prime }+\hbar \omega$ in the conduction band, where *n* denotes the density of states. On the other hand, the electronic structures of insulating materials are similar to semi-conducting materials, except their wider forbidden bands, such that their SEE probability can also be described by Equation (12).

### 2.3. Phonon scattering

The differential energy loss probability for the primary electrons incident into SiO_2_ and for the secondary electrons generated therein is determined by the ELF in Figure 2, where the two energy loss peaks in the range of $\hbar \omega <E_{g}$ are resulted from the interaction of the transport electrons with lattice for the two longitudinal-optical (LO) phonon modes with energies of 63 and 153 meV [77]. The interaction of the transport electrons in ionic crystals for the LO phonons can be described by the Frohlich perturbation theory [100], with which Llacer and Garwin [101] have investigated the transport of electrons with the energy of 0.25–7.5 eV in alkali halides. Based on their work, Fitting and Friemann [102] have derived the creation and annihilation frequencies of the LO phonon in SiO_2_: the LO phonon creation frequency, i.e. the number of LO phonons with an energy ℏω created per unit time by a transport electron with an energy E and effective mass $m^{*}$, is expressed as (in s^−1^),

(14) $$\begin{array}{rl} & f^{+}=\left(n+1\right)\frac{1}{\hbar^{2}}\sqrt{\frac{m^{*}}{2E}}\frac{e^{2}}{4\pi \varepsilon_{0}}\left(\frac{1}{\varepsilon \left(\infty \right)}-\frac{1}{\varepsilon \left(0\right)}\right)\\ & \;\;\;\;\;\;\;\;\;\;\;\hbar \omega \ln\frac{1+\sqrt{1-\left(\hbar \omega /E\right)}}{1-\sqrt{1-\left(\hbar \omega /EE\right)}}, \end{array}$$

where $\varepsilon_{0}$ is the dielectric constant of vacuum, $\varepsilon \left(0\right)$ and $\varepsilon \left(\infty \right)$ are respectively the relative static and optical dielectric constants ($\varepsilon \left(0\right)=3.9$ and $\varepsilon \left(\infty \right)=2.25$ for SiO_2_), and $n=1/\left[\exp\left(\hbar \omega /k_{B}T\right)-1\right]$ is the Bose distribution of the phonon mode population with energy ℏω at temperature *T*. The corresponding phonon annihilation frequency, the number of LO phonons annihilated by a transport electron per unit time, is given by (in s^−1^),

(15) $$\begin{array}{c} f^{-}=n\frac{1}{\hbar^{2}}\sqrt{\frac{m^{*}}{2E}}\frac{e^{2}}{4\pi \varepsilon_{0}}\left(\frac{1}{\varepsilon \left(\infty \right)}-\frac{1}{\varepsilon \left(0\right)}\right)\\ \hbar \omega \ln\frac{1+\sqrt{1+\left(\hbar \omega /E\right)}}{-1+\sqrt{1+\left(\hbar \omega /E\right)}}. \end{array}$$

**Figure 3.**
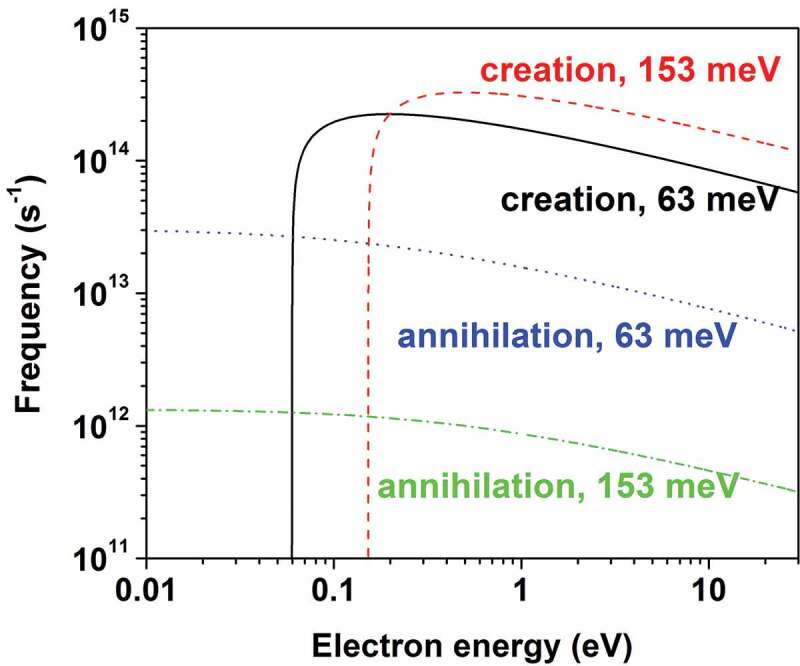
Frequencies of phonon creation and phonon annihilation as functions of electron energy for the 63 and 153 meV LO phonon modes in SiO_2._

Electron-lattice interaction, however, becomes significant only for the electron energy below tens of eV. Figure 3 shows the calculated $f^{+}$ and $f^{-}$as functions of electron energy in the range of 0–30 eV. When the energy is above 153 meV, $f^{+}$ is at least one order of magnitude greater than $f^{-}$ for both LO phonon modes, so that the electron-lattice interaction causes the energy loss for the creation of the LO phonon.

Furthermore, in creating or annihilating a LO phonon, the angular probability for a transport electron to be scattered into polar angle θ within a solid angle dΩ is described by the probability distribution [102],

**Figure 4.**
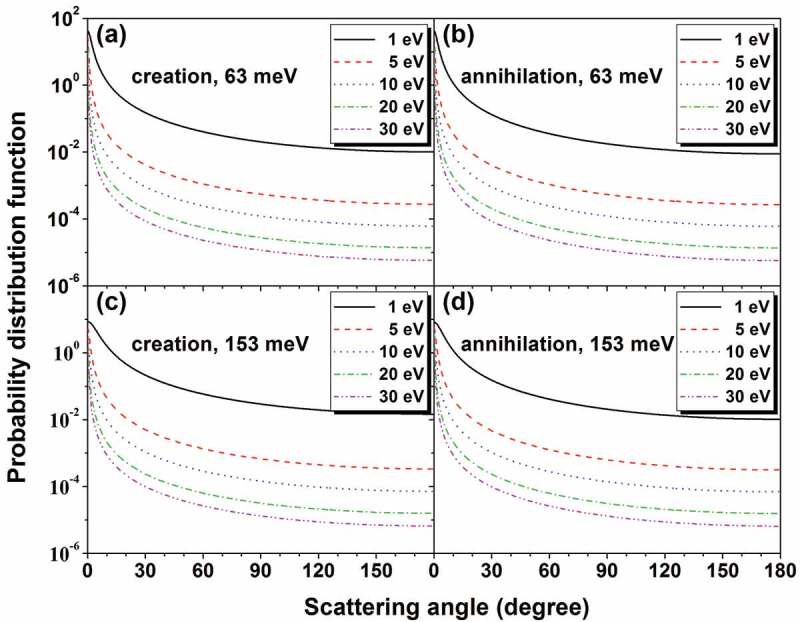
Angular probability distributions at several different electron energies for creation and annihilation of 63 and 153 meV LO phonons in SiO_2._

(16) $$\frac{dw}{d\Omega }=\frac{\frac{1}{E+E^{\prime }-2{\left(EE^{\prime }\right)}^{1/2}\cos\theta }}{\pi {\left(EE^{\prime }\right)}^{-1/2}\ln\frac{E+E^{\prime }+2{\left(EE^{\prime }\right)}^{1/2}}{E+E^{\prime }-2{\left(EE^{\prime }\right)}^{1/2}}},$$

where E and $E^{\prime }$ are, respectively, the electron energy before and after the phonon scattering. Note that $E^{\prime }=E-\hbar \omega$ if a LO phonon is created and $E^{\prime }=E+\hbar \omega$ if a LO phonon is annihilated. In Figure 4, dw/dΩ is plotted as a function of the scattering angle θ for an electron at different energies when 63 and 153 meV LO phonons are created or annihilated. Forward scattering is clearly predominant in Figure 4.

On the other hand, the transport electrons can also interact with acoustic phonons. In contrast to LO phonons, acoustic phonons mainly alter electron moving direction, but not electron energy since the energy of acoustic phonon is quite limited. Thus, acoustic phonon scatterings can be regarded as elastic scattering processes. The creation and annihilation frequencies of acoustic phonons by a transport electron are given by [103]:

(17) $$f_{AC}^{\pm }\left(E\right)=\frac{3m^{*}C_{AC}^{2}}{4\pi \rho \hbar k}\int_{q_{c}}^{q_{\max}}dq\frac{q^{3}}{\hbar \omega_{AC}\left(q\right)}\left(\frac{1}{2}\pm \frac{1}{2}+n_{AC}\right),$$

where $C_{AC}$ is the electron-acoustic phonon coupling constant; $q_{c}=2m^{*}c_{s}$ and $q_{\max}=2k-q_{c}$, where $c_{s}$ is the effective sound velocity obtained by averaging over the longitudinal and transverse branches. The angular frequency of acoustic phonons varies largely with the phonon momentum as described by the dispersion relation [103]:

(18) $$\begin{array}{c} \hbar \omega_{AC}\left(q\right)\\ =\left\{\begin{array}{c} \left(2/\pi \right)\hbar k_{BZ}c_{s}{\left[1-\cos\left(\pi q/2k_{BZ}\right)\right]}^{1/2}\;\left(q<k_{BZ}\right);\\ \left(2/\pi \right)\hbar k_{BZ}c_{s}\;\left(q\geq k_{BZ}\right), \end{array}\right. \end{array}$$

where $k_{BZ}$ is the wave vector at the Brillouin zone boundary.

Different from LO phonons, acoustic phonons mainly affect electrons at very high electric fields. This can be inferred from the fact that the average electron energy in SiO_2_ cannot continuously increase with the electric field exposed on the sample, but stabilizes at about 5 eV when the electric field reaches 10 MV cm^−1^ [104]. The acoustic-phonon scattering is important for this observation that it increases the effective transport length of electron trajectories in the sample, giving rise to the excitation of more LO phonons and to the stabilization of the average electron energy once the energy gain from the electric field is offset by the electron-LO interaction. In addition, this enhancement of LO-phonon excitations due to acoustic-phonon scattering is also responsible for the Si-2p peak broadening in the energy spectrum of the photoelectrons emitted from a SiO_2_ film grown on a Si substrate under photon illumination [105].

However, the $f_{AC}^{\pm }$ calculated by Equation (17) keeps increasing in the electron energy range of $E>E_{g}$ [103], which is not reasonable since it has been found experimentally that the scattering rate of acoustic phonons is reduced as the electron energy increases from 8 to 16 eV in SiO_2_ [105]. To remedy this deficiency, Bradford and Woolf [106] proposed to apply a screened Coulomb potential to the calculation of the total electron-phonon scattering cross section, and the calculated result showed a reduction tendency with the increasing electron energy in the range of $E>E_{g}$.

**Figure 5.**
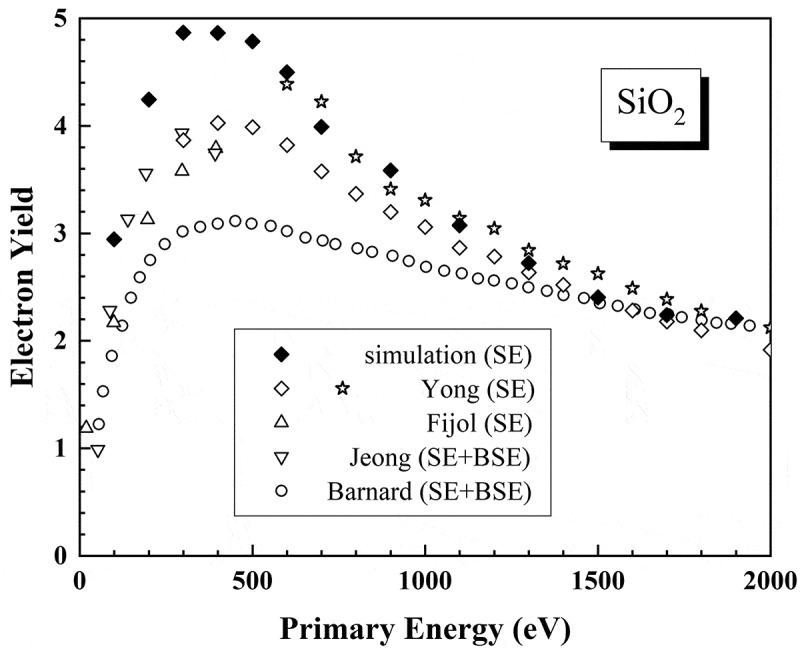
Comparison on the electron yields, secondary electron (SE) yield and total yield as the sum of SE yield and backscattered electron (BSE) coefficient, of SiO_2_ between experiments (open symbols) and a Monte Carlo simulation without considering charging (solid symbols) (update of Figure 3(a) in Ref [98].)

Acoustic phonons are not likely to severely influence SEE in electron beam irradiation experiments because the calculated secondary electron yields of SiO_2_ by a model without considering the acoustic-phonon scattering still agree with an experiment [98] even if not all, as shown in Figure 5, which displays the intrinsic character of secondary electron emission without charging. Similarly, without taking the acoustic-phonon scattering into account the calculated secondary electron yields of CsI under photon illumination by a Monte Carlo method agree also with experiment [107]. In particular, Ganachaud et al. [99] have pointed it out that whether or not taking the acoustic-phonon scattering into account would not cause an obvious variation in the secondary electron yield in their Monte Carlo simulations, but incorporating it into the simulation would result in a narrower secondary electron peak. On the other hand, it was shown that the omit of acoustic-phonon scattering results in a significant overestimation of secondary electron yield only for primary energies below 200 eV in a model sensitivity analysis of Monte Carlo simulation of SEM image contrast [108].

## 3. Computation of potential

With the theoretical framework described in Section 2, the transport electrons can be traced until they form deposited charges, i.e. the polarons, when their energies are exhausted. As has been mentioned previously, the polarons, including not only the energy-exhausted electrons but also the holes, cannot maintain stationary but will drift under the electric field. The charge drift is described by the drift velocity, which determines the drift speed and direction and can be calculated by multiplying the charge mobility and the electric field.

However, the drifting charges can be trapped by the trapping sites on their drift paths, which plays a fundamental role in the formation of the charging effect. To take the charge trapping into account, one usually assumes a uniform distribution of trapping sites in the sample. Once a charge is drifted to a trapping site, three kinds of fate may occur [98,109–111]: (1) if the trapping site is empty, the drifting charge will be trapped there; (2) if the trapping site has been occupied by a charge with an opposite sign to that of the drifting charge, these two charges will recombine with each other and this trapping site becomes empty; (3) if the trapping site has been occupied by a charge with the same sign as that of the drifting charge, the drifting charge will continue to drift until it is trapped or recombined elsewhere.

With the above assumptions, one can obtain the spatial distribution of the trapped charges in the sample by a Monte Carlo simulation and the potential distribution generated inside and outside the sample. Since the potential distribution is indispensable for simulating charging effect, we hereby introduce several methods that can be employed, and by differentiating potential distribution one further drives directly the electric field distribution. The charging effect is a time-dependent phenomenon, due essentially to the time variation of the trapped charge distribution under the electron beam irradiation. Therefore, the trapped charge distribution is time dependent and so does the potential distribution. It has to note that the following methods are for calculating the potential distribution at a certain time instant and none of them takes time as a variable. However, in simulating charging effect, the potential calculation with any method should be repeated regularly during the electron beam irradiation period to update the time-varying potential distribution.

### 3.1. Image-charge method

Polarized charges can be present on interfaces or surfaces of the insulating sample and contribute partially to the potential distribution. However, the distribution of these polarized charges is hard to know, increasing the difficulty of calculating the potential distribution. The image-charge method uses the image charges to replace the polarized charges on interfaces or surfaces in the way that the potential distribution is generated jointly by the real trapped charges and the image charges intentionally set in the space. Setting the image charges, including their positions and charge quantities, is easy for simple structural geometry, such as the semi-infinite and film/substrate structures, but is very difficult for complex structure morphology. Importantly, the potential distribution calculated by using the image-charge method has to satisfy the boundary condition for insulating samples:

(19) $$e_{n}\cdot \left(D_{1}-D_{2}\right)=0,$$

where $D_{1,2}=\varepsilon_{1,2}E_{1,2}$ is the electric displacement vector, with $\varepsilon_{1,2}$ being the respective dielectric constants of the two adjacent media and $E_{1,2}$ the respective electric field vectors close to the interface, and $e_{n}$ is the unit vector perpendicular to the interface.

#### 3.1.1. Mono-image-charge method

The mono-image-charge method [98] applies only to a semi-infinite sample, for which one image charge should be set for each free charge. Let a free charge with charge quantity $q_{f}$ be located at $r_{f}=\left(x_{0},y_{0},z_{0}\right)$ inside the sample $\left(z_{0}<0\right)$, then the potential at a point $r=\left(x,y,z\right)$ is calculated as,

(20) $$\phi \left(r\right)=\frac{q_{f}}{4\pi \varepsilon |r-r_{f}|}+\frac{q_{i}}{4\pi \varepsilon_{0}|r-r_{i}|},$$

where ε is the dielectric constant of the sample and $q_{i}$ is the charge quantity of the image charge set at $r_{i}=\left(x_{i},y_{i},z_{i}\right)$. In particular, $q_{i}$ and $r_{i}$ can be ascertained by requiring the potential in Equation (20) to satisfy the boundary condition at the sample surface described by Equation (19). Specifically, $q_{i}$ is given by

(21) $$q_{i}=\frac{\varepsilon_{0}\left(\varepsilon -\varepsilon_{0}\right)}{\varepsilon \left(\varepsilon +\varepsilon_{0}\right)}q_{f}.$$

However, $r_{i}$ depends not only on $r_{f}$ but also on r: if r is located in vacuum (z>0), $r_{i}=r_{f}$; otherwise, $r_{i}=\left(x_{0},y_{0},-z_{0}\right)$.

#### 3.1.2. Multi-image-charge method

The multi-image-charge method [98] presented here applies to a sandwich structure containing three layers with the respective dielectric constants of $\varepsilon_{1}$, $\varepsilon_{2}$, and $\varepsilon_{3}$. In particular, the first, second, and third layers can be regarded as the vacuum, film, and substrate, respectively. However, the polarized charges distributed on an interface can interact with those distributed on the other interface, requiring more image charges to be set for each free charge. Meanwhile, the potential calculation depends also on the location of a free charge.

If the free charge is in the film, the second layer, the potential at r can be calculated as,

(22) $$\phi \left(r\right)=\frac{q_{f}}{4\pi \varepsilon_{2}|r-r_{f}|}+\sum_{i}\frac{q_{i}}{4\pi \varepsilon_{0}|r-r_{i}|},$$

where the sum runs over all the image charges. Similar to the mono-image method, the image charges here are set by requiring the potential calculated using Equation (22) to satisfy the boundary condition, Equation (19). Table 1(a) shows the corresponding charge quantities and positions of the image charges, with *a* representing the vertical distance from the free charge to the interface between the second and third layers, and *b* the vertical distance from the free charge to the interface between the first and second layers. In Table 1(a), four groups of image charges are introduced for each free charge depending on the position where the potential is calculated, with *n* = 1, 2 … 8. The horizontal coordinates, *x* and *y*, of all the image charges are the same as those of the free charge. The parameters $\beta_{1}=\left(\varepsilon_{2}-\varepsilon_{3}\right)/\left(\varepsilon_{2}+\varepsilon_{3}\right)$ and $\beta_{2}=\left(\varepsilon_{2}-\varepsilon_{1}\right)/\left(\varepsilon_{2}+\varepsilon_{1}\right)$ are related to the dielectric constants; $q_{0}=q_{f}\varepsilon_{0}/\varepsilon_{2}$ is the net charge, which is the sum of the free charge $q_{f}$ and its neighboring polarized charges, which have the opposite sign to that of the free charge; and c=a+b is the thickness of the second layer.

**Table 1.** Image-charge quantities $q_{i}$ and vertical positions $z_{i}$ for a structure consisting of three layers with respective dielectric constants $\varepsilon_{1}$, $\varepsilon_{2}$, and $\varepsilon_{3}$ when the free charge is inside (a) the second layer or (b) the third layer. The horizontal coordinates $\left(x_{i},y_{i}\right)$ of each image charge are the same as those of the free charge $\left(x_{0},y_{0}\right)$, i.e. $\left(x_{i},y_{i}\right)=\left(x_{0},y_{0}\right)$. Note that z=0 is taken as the vertical coordinate of the interface between the first and second layers; z<0 and z>0 represent the regions below and above this interface, respectively. Four sets of image charges are given, and n=1,2⋅⋅⋅8 for each set; therefore, a total of 32 image charges are necessary for each free charge in each case. Reprinted from Ref. [98] with permission from IOP publishing. © IOP Publishing. Reproduced with permission. All rights reserved

Medium of field position interested	$q_{i}$	$z_{i}$	
(a) the free charge is located inside the second medium $\varepsilon_{2}$			
$\varepsilon_{1}$	$\beta_{1}{\left(\beta_{1}\beta_{2}\right)}^{n-1}q_{0}$	$-a-\left(2n-1\right)c$	
	$\beta_{2}{\left(\beta_{1}\beta_{2}\right)}^{n-1}q_{0}$	$-b-\left(2n-2\right)c$	
	${\left(\beta_{1}\beta_{2}\right)}^{n}q_{0}$	$-a-\left(2n-1\right)c$	
	${\left(\beta_{1}\beta_{2}\right)}^{n}q_{0}$	−b−2nc	
$\varepsilon_{2}$	$\beta_{1}{\left(\beta_{1}\beta_{2}\right)}^{n-1}q_{0}$	$-a-\left(2n-1\right)c$	
	$\beta_{2}{\left(\beta_{1}\beta_{2}\right)}^{n-1}q_{0}$	$\;\;b+\left(2n-2\right)c$	
	${\left(\beta_{1}\beta_{2}\right)}^{n}q_{0}$	a+(2n−1)c	
	${\left(\beta_{1}\beta_{2}\right)}^{n}q_{0}$	−b−2nc	
$\varepsilon_{3}$	$\beta_{1}{\left(\beta_{1}\beta_{2}\right)}^{n-1}q_{0}$	$\;\;a+\left(2n-3\right)c$	
	$\beta_{2}{\left(\beta_{1}\beta_{2}\right)}^{n-1}q_{0}$	$\;\;b+\left(2n-2\right)c$	
	${\left(\beta_{1}\beta_{2}\right)}^{n}q_{0}$	a+(2n−1)c	
	${\left(\beta_{1}\beta_{2}\right)}^{n}q_{0}$	$\;\;b+\left(2n-2\right)c$	
(b) the free charge is located inside the third medium $\varepsilon_{3}$			
$\varepsilon_{1}$	$\beta_{1}{\left(\beta_{1}\beta_{2}\right)}^{n-1}q_{0}$		$-b-\left(2n-2\right)c$
	$\beta_{2}{\left(\beta_{1}\beta_{2}\right)}^{n-1}q_{0}$		$-b-\left(2n-2\right)c$
	${\left(\beta_{1}\beta_{2}\right)}^{n}q_{0}$		$-b-\left(2n-2\right)c$
	${\left(\beta_{1}\beta_{2}\right)}^{n}q_{0}$		−b−2nc
$\varepsilon_{2}$	$\beta_{1}{\left(\beta_{1}\beta_{2}\right)}^{n-1}q_{0}$		$-b-\left(2n-2\right)c$
	$\beta_{2}{\left(\beta_{1}\beta_{2}\right)}^{n-1}q_{0}$		$\;\;b+\left(2n-2\right)c$
	${\left(\beta_{1}\beta_{2}\right)}^{n}q_{0}$		b+(2n−2)c
	${\left(\beta_{1}\beta_{2}\right)}^{n}q_{0}$		−b−2nc
$\varepsilon_{3}$	$\beta_{1}{\left(\beta_{1}\beta_{2}\right)}^{n-1}q_{0}$		$\;\;b+\left(2n-4\right)c$
	$\beta_{2}{\left(\beta_{1}\beta_{2}\right)}^{n-1}q_{0}$		$\;\;b+\left(2n-2\right)c$
	${\left(\beta_{1}\beta_{2}\right)}^{n}q_{0}$		b+(2n−2)c
	${\left(\beta_{1}\beta_{2}\right)}^{n}q_{0}$		$\;\;b+\left(2n-2\right)c$

If the free charge is in the third layer, the potential at r can be calculated as

(23) $$\phi \left(r\right)=\frac{q_{f}}{4\pi \varepsilon_{3}|r-r_{f}|}+\sum_{i}\frac{q_{i}}{4\pi \varepsilon_{0}|r-r_{i}|}.$$

The corresponding image-charge setting is given in Table 1(b). Four groups of image charges are also introduced for this case, with *n* = 1, 2 … 8. The parameters here are $\beta_{1}=-\left(\varepsilon_{2}-\varepsilon_{3}\right)/\left(\varepsilon_{2}+\varepsilon_{3}\right)$,$\beta_{2}=\left(\varepsilon_{2}-\varepsilon_{1}\right)/\left(\varepsilon_{2}+\varepsilon_{1}\right)$, and $q_{0}=q_{f}\varepsilon_{0}/\varepsilon_{3}$; *a* is the vertical distance from the free charge to the interface between the second and third layers, *b* is the vertical distance from the free charge to the interface between the first and second layers, and c=b−a is the thickness of the second layer.

### 3.2. Finite-difference method

By the finite difference method [99] the Poisson equation,

(24) $$\nabla \cdot \left[\varepsilon \left(r\right)\nabla \phi \left(r\right)\right]=-\rho \left(r\right),$$

is discretized at first. For a homogeneous medium the discretized Poisson equation with a regular cubic spatial mesh is

(25) $$\begin{array}{rl} & \phi_{i-1,j,k}+\phi_{i+1,j,k}+\phi_{i,j-1,k}+\phi_{i,j+1,k}+\phi_{i,j,k-1}\\ & +\phi_{i,j,k+1}-6\phi_{i,j,k}=-\rho_{i,j,k}u^{2}/\varepsilon , \end{array}$$

where ϕ is the potential, ρ the charge density, *u* the mesh size, and ε the dielectric constant of the medium. The subscript set (*i, j, k*) represents a mesh point in Cartesian coordinate system.

For a semi-infinite sample, the boundary condition, Equation (19), can be expressed by writing the surface potential $\phi_{i,j,0}$ as

(26) $$\phi_{i,j,0}=\frac{\varepsilon_{0}\phi_{i,j,1}+\varepsilon \phi_{i,j,-1}}{\varepsilon_{0}+\varepsilon },$$

where (*i, j*, 1) and (*i, j*, – 1) are the mesh points near the sample surface and are located in vacuum and in the sample, respectively.

However, if an irregular non-cubic mesh is used, the discretized Poisson equation can be written as

(27) $$\begin{array}{c} \frac{1}{\left|x_{i+1,j,k}-x_{i,j,k}\right|+\left|x_{i,j,k}-x_{i-1,j,k}\right|}\\ \left\{\frac{\phi_{i+1,j,k}-\phi_{i,j,k}}{\left|x_{i+1,j,k}-x_{i,j,k}\right|}-\frac{\phi_{i,j,k}-\phi_{i-1,j,k}}{\left|x_{i,j,k}-x_{i-1,j,k}\right|}\right\}+\\ \frac{1}{\left|y_{i,j+1,k}-y_{i,j,k}\right|+\left|y_{i,j,k}-y_{i,j-1,k}\right|}\\ \left\{\frac{\phi_{i,j+1,k}-\phi_{i,j,k}}{\left|y_{i,j+1,k}-y_{i,j,k}\right|}-\frac{\phi_{i,j,k}-\phi_{i,j-1,k}}{\left|y_{i,j,k}-y_{i,j-1,k}\right|}\right\}+\\ \frac{1}{\left|z_{i,j,k+1}-z_{i,j,k}\right|+\left|z_{i,j,k}-z_{i,j,k-1}\right|}\\ \left\{\frac{\phi_{i,j,k+1}-\phi_{i,j,k}}{\left|z_{i,j,k+1}-z_{i,j,k}\right|}-\frac{\phi_{i,j,k}-\phi_{i,j,k-1}}{\left|z_{i,j,k}-z_{i,j,k-1}\right|}\right\}=-\frac{\rho_{i,j,k}}{2\varepsilon }, \end{array}$$

where *x, y*, and *z* are the coordinates of a mesh point. Meanwhile, Equation (26) is expressed as

(28) $$\phi_{i,j,0}=\frac{\varepsilon_{0}\phi_{i,j,1}\left|x_{i,j,0}-x_{i,j,-1}\right|+\varepsilon \phi_{i,j,-1}\left|x_{i,j,1}-x_{i,j,0}\right|}{\varepsilon_{0}\left|x_{i,j,0}-x_{i,j,-1}\right|+\varepsilon \left|x_{i,j,1}-x_{i,j,0}\right|}.$$

Note that when the irregular non-cubic mesh is replaced by the regular cubic mesh, Equations (27) and (28) are reduced to Equations (25) and (26), respectively.

In application of finite difference method to a sample made of complex 3D structures, the dielectric constant in the general form of Poisson equation, Equation (24), is spatial varying, but, could be a constant within a structure volume. The discretization of Equation (24) has a different form from Equation (25) at an interface between two mediums contained in different structures [112]. The simultaneous linear equations for the potentials at discretized grid locations should be solved under the Neumann boundary condition on the interface and Dirichlet boundary condition at the specified locations. The solution requires to compute a large matrix.

### 3.3. Finite element analysis

For complex structures, the finite element analysis (FEA) can be used to calculate potential distribution by following a four-step procedure [113]. The first step is to divide the space, where the potential distribution is desired, into a group of subregions, and each subregion is actually an element. The second step is to derive the potential expression for each element from the potentials at its vertices with the aid of a chosen interpolation function. The third step is to obtain, with the application of the Ritz method or the Galerkin method, a set of equations taking the potentials at the vertices of all elements as variables. The fourth step is to obtain the potentials at all vertices by solving the set of equations obtained in the third step.

However, in limited-boundary problems, the potential distribution is governed by the combination of the Poisson equation and the boundary condition. Ref. [113] introduces how the boundary conditions, such as the Dirichlet condition, are incorporated into FEA when solving a general differential equation. For the charging effect, the typical boundary condition at the insulator-vacuum interface or the insulator-insulator interface has been given by Equation (19), while that for the metallic regions contained in a sample will be introduced in Section 4.3.2.

### 3.4. Self-consistent method

The self-consistent method [98] is another useful method to calculate the potential distribution for complex structures. At first, it has to be clear that both the free charges, which mean the trapped charges here, and the polarized charges can contribute to the potential distribution. The polarized charges are distributed either in the vicinity of the free charge or on an interface between two media. By combining the free charge with the polarized charges surrounding it, the Poisson equation is written as

(29) $$\nabla^{2}\phi \left(r\right)=-\left(\frac{\rho_{f}}{\varepsilon }+\frac{\rho_{p}}{\varepsilon_{0}}\right),$$

where $\rho_{f}$ and $\rho_{p}$ are, respectively, the charge densities of the free charge in the sample and of the polarized charges on the interface. The self-consistent method is to separately calculate the potential distribution generated by the free charge and that by the polarized charges on the interface.

Firstly, the potential generated by the free charge is calculated as

(30) $$\phi_{f}\left(r\right)=\int \frac{\rho_{f}\left(r{\;}^{\prime }\right)}{4\pi \varepsilon \left(r^{\prime }\right)|r-r{\;}^{\prime }|}\;dr{\;}^{\prime }.$$

For the potential due to the polarized charge distribution on the interface it is crucial to know the distribution, which is given by

(31) $$\rho_{p}=-e_{n}\cdot \left(P_{2}-P_{1}\right),$$

where $P_{1,2}=\left(\varepsilon_{1,2}-\varepsilon_{0}\right)E_{1,2}$ is the polarization density in medium $\varepsilon_{1,2}$ under the electric field $E_{1,2}$, and $e_{n}$ is the unit vector perpendicular to the interface and pointing from medium $\varepsilon_{1}$ to medium $\varepsilon_{2}$. Then, the potential generated by the polarized charge on the interface can be given by

(32) $$\phi_{p}\left(r\right)=-Q_{p-f}\int \frac{\rho_{p}\left(r{\;}^{\prime }\right)}{4\pi \varepsilon_{0}|r-r{\;}^{\prime }|\int \rho_{p}\left(r^{\prime \prime }\right)\;dr^{\prime \prime }}\;dr{\;}^{\prime },$$

where $Q_{p-f}=-\left(1-\varepsilon_{0}/\varepsilon \right)\int \rho_{f}\left(r\right)\;dr$ is the total quantity of the polarized charge surrounding the free charge, and ε is the dielectric constant of the sample where the free charge is located in.

Finally, the desired total potential is obtained via the sum of two parts,

(33) $$\phi \left(r\right)=\phi_{f}\left(r\right)+\phi_{p}\left(r\right).$$

In the self-consistent calculation, the first step is to use Equation (30) to calculate the potential generated by the free charge. Then, Equation (31) is used to obtain the polarized charge distribution on the interface using the free-charge-generated electric field $E\approx -\nabla \phi_{f}\left(r\right)$ and polarization density $P_{1,2}=\left(\varepsilon_{1,2}-\varepsilon_{0}\right)E_{1,2}$. Next, Equation (32) is used to obtain the potential generated by these polarized charges, after which the total potential $\phi \left(r\right)$ can be found via Equation (33). However, the electric field involved in Equation (31) should be the total one, $E=-\nabla \phi \left(r\right)$. Thus, the obtained total potential needs to be differentiated, and this total electric field is then substituted into Equation (31) to update the polarized charge distribution. Once the polarized charge distribution changes, the potential it generates also changes, and so does the total potential. The calculation therefore needs to be iterated until the potential distribution converges.

**Figure 6.**
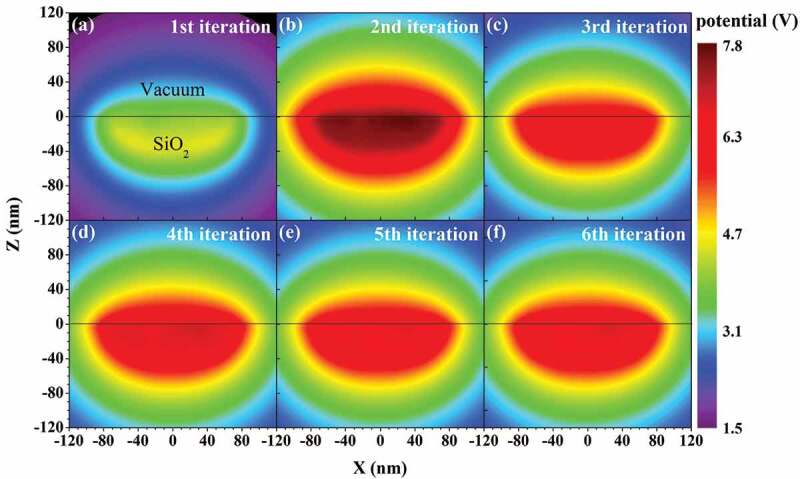
Convergence of the simulated potential distribution in the self-consistent method. An electron beam with a primary energy of 0.5 keV, beam size of 100 nm, and beam current of 0.16 nA is normally incident into the semi-infinite SiO_2_ structure

**Figure 7.**
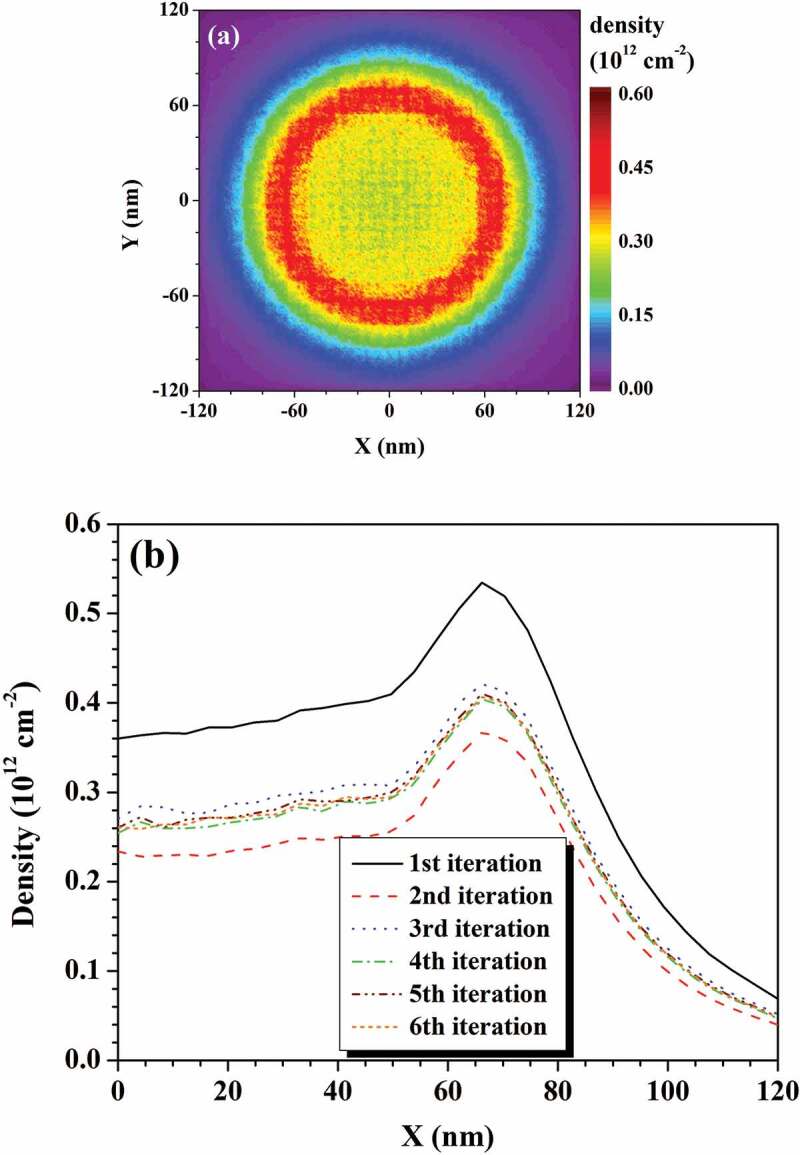
(a) Polarized charge distribution on the surface of the semi-infinite SiO_2_ structure obtained at the 6th iteration. Reprinted from Ref. [98] with permission from IOP publishing. © IOP Publishing. Reproduced with permission. All rights reserved. (b) Convergence of the polarized charge distribution. The simulation conditions are the same as in Figure 6

To see the convergence in this self-consistent method intuitively, Figure 6 shows the evolution of the potential distribution with iteration for the charging effect of a semi-infinite SiO_2_ sample. In the simulation, an electron beam with a primary electron energy of 0.5 keV, beam current of 0.16 nA, and beam size of 100 nm is normally incident into the sample. Initially, there is an obvious potential variation between successive iterations, but as the iteration goes on, the potential change decreases until the final convergence is reached by the 5th iterations. Figure 7 shows the evolution of the polarized charge distribution on the surface with iteration. The polarized charges are symmetrically distributed with respect to the incidence position of the primary electron beam. Since the polarization density of vacuum is always zero, the distribution of the polarized charges in Figure 7 is proportional to the polarization density just beneath the sample surface. Furthermore, the polarization density at a particular position in the sample is proportional to the electric field there. Thus, both the polarization density and electric field have the same distribution near the surface as that of the polarized charges in Figure 7.

## 4. Charging effect simulation

This section is to present some quantitative characteristics of the charging effect by reviewing the related Monte Carlo simulations and experiments. It is worth noting that the advantage owned by the Monte Carlo method over other theoretical methods in simulating the charging effect relies essentially on the fact that it can accurately trace electron transport in solid samples by including both incident electrons and cascade secondary electrons with the updated Monte Carlo model [72,97,98]. This is achieved at the cost of considerable computation time; for the CTMC-CHARG model simulation [114] with the self-consistent method the typical CPU time is about tens of hours on a parallel computer and the memory required is about several GB [98].

**Figure 8.**
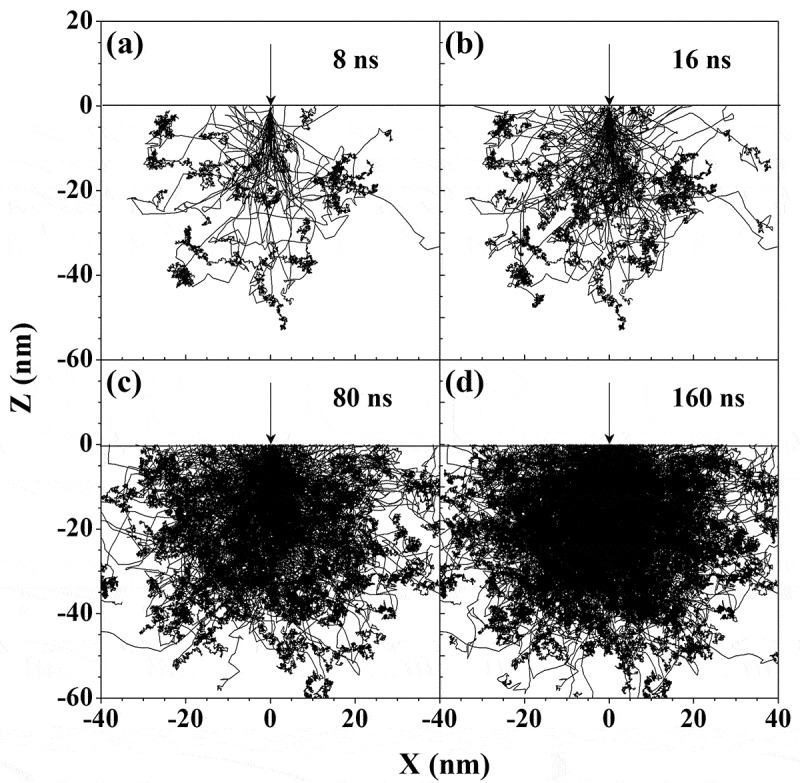
Simulated trajectories of primary electrons in SiO_2_ for an electron beam with energy of 1 keV and current of 1 nA normally incident into a semi-infinite SiO_2_ sample. The trajectories are from *t* = 0 (the beginning of primary electron incidence) to (a) 8, (b) 16, (c) 80, and (d) 160 ns, respectively

Before starting with the discussion on the charging effect, it is shown in Figure 8 the primary electron trajectories simulated for the 1 keV incident electrons into a semi-infinite SiO_2_ sample according to the framework detailed in Section 2 without considering the charging effect. It can be seen that the electron trajectories representing electron motion and scattering are rather complex, and the trajectory distribution is getting denser as time goes on, but remains symmetrical about the beam incidence direction.

### 4.1. Categories of charging effect

Take the charging effect of an insulating foil induced by the irradiation of a normally incident primary electron beam as an example [115], in which the foil of a certain thickness is bounded on the top side by vacuum and on the bottom side by a metallic substrate, the charging effect is simplified to a one-dimensional problem by taking the primary electron beam to be defocused, where the following continuity equation should be satisfied:

(34) $$\begin{array}{rl} & J_{0}=J_{0}\delta \left(t\right)+J_{0}\eta \left(t\right)\pm J_{s}\left(t\right)-\partial \left[Q\left(t\right)\right]/\partial t,\\ & \;\;\;\;=J_{0}\sigma \left(t\right)\pm J_{s}\left(t\right)-\partial \left[Q\left(t\right)\right]/\partial t \end{array}$$

where $J_{0}$ is the current density of the primary electron beam, δ the secondary electron yield, η the backscattering coefficient, σ the total electron yield, $J_{s}$ the leakage current, and Q the net quantity of the charges accumulated in the foil. To further simplify the problem, we neglect $J_{s}$, implying the foil thickness is much greater than the penetration depth of the primary electron beam; Equation (34) then becomes:

(35) $$J_{0}=J_{0}\sigma \left(t\right)-\partial \left[Q\left(t\right)\right]/\partial t.$$

In particular, the quantity of positive charges accumulated in the foil can be given by

(36) $$Q_{+}\left(t\right)=J_{0}\int_{0}^{t}\delta \left(t^{\prime }\right)\;\;dt^{\prime },$$

and that of negative charges by

(37) $$Q_{-}\left(t\right)=-J_{0}\int_{0}^{t}\left[1-\eta \left(t^{\prime }\right)\right]\;\;dt^{\prime }.$$

Thus, the total quantity of the accumulated charges is

(38) $$\begin{array}{rl} & Q\left(t\right)=Q_{+}\left(t\right)+Q_{-}\left(t\right)\\ & \;\;\;\;\;=J_{0}\int_{0}^{t}\left[\delta \left(t^{\prime }\right)+\eta \left(t^{\prime }\right)-1\right]\;\;dt^{\prime }\\ & \;\;\;\;\;=J_{0}\int_{0}^{t}\left[\sigma \left(t^{\prime }\right)-1\right]\;\;dt^{\prime } \end{array}$$

In the steady state of the charging condition at enough long time, $\partial \left[Q\left(+\infty \right)\right]/\partial t=0$, so $\sigma \left(+\infty \right)=1$ according to Equation (35). As will be shown later that for adequately thick samples the total electron yield usually varies in a monotonous manner in the charging effect, either decreasing from a greater value down to the unity or increasing from a smaller value up to the unity. Thus, the total electron yield at the very beginning of the irradiation, $\sigma_{0}$, which is actually the value without the charging effect, essentially determines the sign of the net quantity of the accumulated charges and that of the charging effect: if $\sigma_{0}>1$, Q>0 and the charging effect is positive; if $\sigma_{0}<1$, Q<0 and the charging effect is negative; if $\sigma_{0}=1$, Q=0 and the charging effect is absent.

**Figure 9.**
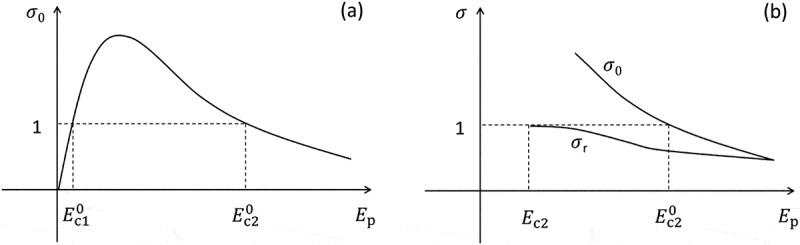
(a) Schematic curve of the total electron yield $\sigma_{0}$ at the very beginning of primary electron irradiation as a function of the primary electron energy $E_{p}$. $E_{c1}^{0}$ and $E_{c2}^{0}$ are the first and second critical primary electron energies, respectively, where $\sigma_{0}=1$. (b) After taking into account the trapping of the secondary electrons and backscattered electrons in the sample on their emission paths, the $\sigma_{0}$-curve is reduced to the $\sigma_{r}$-curve, and the second critical primary electron energy is shifted from $E_{c2}^{0}$ to $E_{c2}^{\;}$

Physically, $\sigma_{0}$ depends strongly on the primary electron energy $E_{p}$, as shown by the schematic $\sigma_{0}$ curve as a function of $E_{p}$ in Figure 9(a). Based on the $\sigma_{0}$ curve, positive charging effect occurs in the range of $E_{c1}^{0}<E_{p}<E_{c2}^{0}$, negative charging effect occurs in the range of $E_{p}<E_{c1}^{0}$ or $E_{p}>E_{c2}^{0}$, and the charging effect is absent at $E_{p}=E_{c1}^{0}$ or $E_{p}=E_{c2}^{0}$, where $E_{c1}^{0}$ and $E_{c2}^{0}$ are the first and second critical primary electron energies, respectively, at which $\sigma_{0}=1$. This prediction manner is often referred to as the total electron yield approach. However, in practical applications of this approach based on the $\sigma_{0}$-curve, errors have been frequently encountered that several predicted positive charging effect in the range of $E_{c1}^{0}<E_{p}<E_{c2}^{0}$ have been experimentally verified to be negative (see the references in Ref [115].). Cazaux [115] has interpreted this deviation by taking into account the trapping of the secondary and backscattered electrons in the sample on their emission paths, due to which the initial $\sigma_{0}$-curve evolves into the reduced $\sigma_{r}$-curve and the second critical primary electron energy shifts from $E_{c2}^{0}$ to $E_{c2}^{\;}$, as shown in Figure 9(b). With this consideration, the incorrectly predicted positive charging effect based on the $\sigma_{0}$-curve can transform to negative based on the $\sigma_{r}$-curve. This implies that the total electron yield approach still works but its application should be based on the $\sigma_{r}$-curve. On the other hand, insulating materials usually have great electron yields, which is resulting not only from the reduced electron-electron interactions due to the lack of inelastic channels above Fermi energy in insulating materials but also from their small surface emission barrier, i.e. the affinity. In consequence, for most insulating materials, $E_{c2}^{0}$ is usually close to or greater than 10 keV [38], while $E_{c2}^{\;}$ is largely reduced and ranges 1–3 keV [28,38].

It is worth mentioning that the curves in Figure 9 are by default for the situations where primary electron beams are normally incident into the semi-infinite samples. The charging effects to be discussed in the following are all induced by the normal irradiation by a primary electron beam, unless otherwise stated.

### 4.2. Characteristics of charging effect

#### 4.2.1. Positive charging effect

To clearly show the basic characteristics of positive charging effect, we at first consider a Monte Carlo simulation of the charging effect of a semi-infinite SiO_2_ sample under the irradiation of a 0.1 keV primary electron beam. In the simulation, the transport of electrons is traced by describing their encountered elastic and inelastic scattering separately. Specifically, the elastic scattering is described by a differential cross-section calculated with a partial-wave expansion method, and, the inelastic scattering by a dielectric functional approach. Besides, the interactions of the transporting electrons with LO phonons and the longitudinal and transverse acoustic (LTA) phonons are taken into account; the former interaction is described by the Frohlich perturbation theory [100], and the latter by the approach proposed by Bradford and Woolf [106] and its extension by Akkerman et al. [116]. Furthermore, a phenomenological formula is used to describe the probability of a low-energy electron to form a polaron in moving a unit path. In addition to the low-energy electrons, holes are regarded as polarons as soon as they are generated. Then, the simulation traces the drift of the polarons in the electric field until they are trapped or recombined when encountering the uniformly set trapping sites according to the three occasions detailed in Section 3. With the procedure mentioned above, the simulation can yield a charge distribution based on which the potential distribution is calculated by using the finite difference method detailed in Section 3.2.

**Figure 10.**
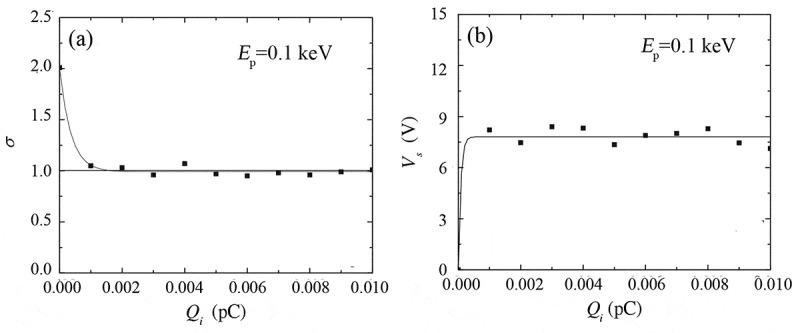
Simulated total electron yield (a) and surface potential (b) as a function of the injected primary electron dose $Q_{i}$ for a semi-infinite SiO_2_ sample. In the simulation, the primary electron energy was taken as 0.1 keV and the trapping site density as 1 × 10^19^ cm^−3^. Adapted from Ref. [111] with permission from John Wiley & Sons, Inc

Figure 10(a) shows the simulated total electron yield as a function of the incident primary electron dose, in which the initial total electron yield is ~2, causing the net accumulation of positive charges. Naturally, the accumulated charges generate a positive surface potential whose evolution is given in Figure 10(b). This positive surface potential, however, behaves like an extra barrier, so that the barrier to be overcome for a secondary electron in its emission process is increased from χ to $\chi +eV_{s}$ in positive charging effect, where $V_{s}$ is the surface potential at the emission site. This barrier impedes secondary electrons from escaping the sample, which is responsible for the reduction of the total electron yield in Figure 10(a). Due to this self-modulation mechanism, the steady state of positive charging effect can be reached; the steady total electron yield is the unity in Figure 10(a) and the steady surface potential is about several volts in Figure 10(b) due to the low energy of secondary electrons.

**Figure 11.**
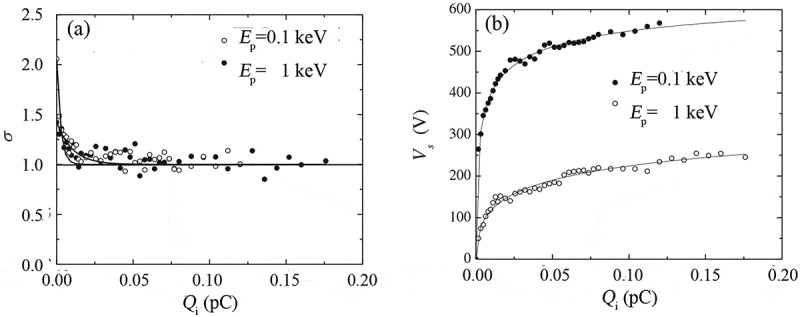
Simulated total electron yield (a) and surface potential (b) as a function of the incident primary electron dose $Q_{i}$ for a semi-infinite SiO_2_ sample . In the simulation, the primary electron energy was taken as 0.1 keV or 1 keV and the trapping site density as 1 × 10^19^ cm^−3^, and a strong extraction potential was assumed to be applied to the electron detector. Adapted from Ref. [111] with permission from John Wiley & Sons, Inc

The discussions presented above apply to the situation that the electron detector is not biased. However, the situation is changed when a strong extraction potential is applied to the electron detector, as illustrated in Figure 11 [111] for the simulated total electron yield and surface potential as a function of the incident dose for a semi-infinite SiO_2_ sample when irradiated by a 0.1 or 1 keV primary electron beam. Figure 11(a) is similar to Figure 10(a), indicating the involved charging effect is still positive, but the surface potential in Figure 11(b) is about two orders of magnitude greater than that in Figure 10(b). This is not difficult to understand that the applied extraction potential facilitates the emission and detection of the low-energy secondary electrons, resulting in the accumulation of more positive charges in the sample and hence a stronger positive surface potential. However, with the accumulation of more and more positive charges, the impediment induced by the positive surface potential will sooner or later exceed the facilitation induced by the extraction potential; once that occurs, the total electron yield in Figure 11(a) begins to decrease until the steady state is reached, so the positive bias applied to the detector delays the arrival of the steady state.

**Figure 12.**
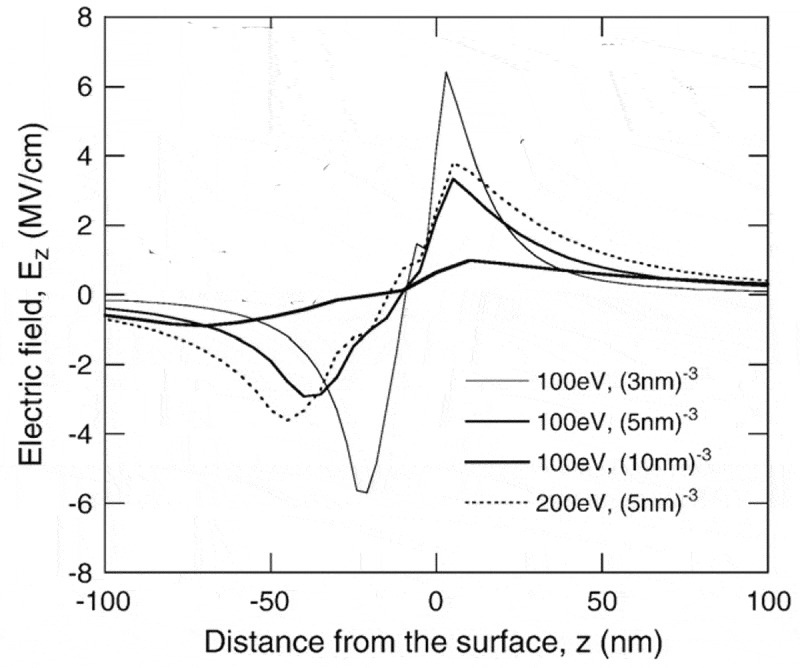
Simulated distribution of the electric field component along the sample surface normal, i.e. the *z*-component, in the steady state of the charging effect for a semi-infinite SiO_2_ sample. The sample (*z* < 0) is bounded by its surface (*z* = 0), above which is vacuum (*z* > 0). Positive values denote that the *z*-component points upwards, while negative values denote that the *z*-component points downwards. The primary electron energies and trapping site densities used in the simulation are indicated in the figure. Adapted from Ref. [109] with permission from Elsevier

It is worth noting that the impediment of SEE by the positive surface potential is just a simplified picture and what really occurs in positive charging effect is the attraction of a portion of low-energy secondary electrons back to the sample surface by the electric field. Figure 12 shows the simulated distribution of the *z*-component of the electric field, i.e. the component along the sample surface normal, in the steady state of the charging effect for a semi-infinite SiO_2_ sample [109]. The Monte Carlo model used in this simulation is quite similar to that used for obtaining Figures 10–11, except that it does not take into account the electron-acoustic phonon interactions but corrects due to the electric field the electron trajectories of primary and secondary electrons in the sample and those of the emitted secondary electrons in vacuum.

The charging effect in Figure 12 has been validated to be positive by the authors via their obtained similar total electron yield and surface potential to Figure 10. Importantly, the *z*-component of the electric field in vacuum (*z* > 0 nm) points upwards (positive values) and maximizes at a certain distance to the surface, and it is mainly this electric field component that attracts the low-energy secondary electrons back to the sample surface. Furthermore, despite the increase of its strength with the increasing trapping site density due to the denser charge distribution, the field is generally in the order of MV cm^−1^ [98,117].

#### 4.2.2. Negative charging effect

**Figure 13.**
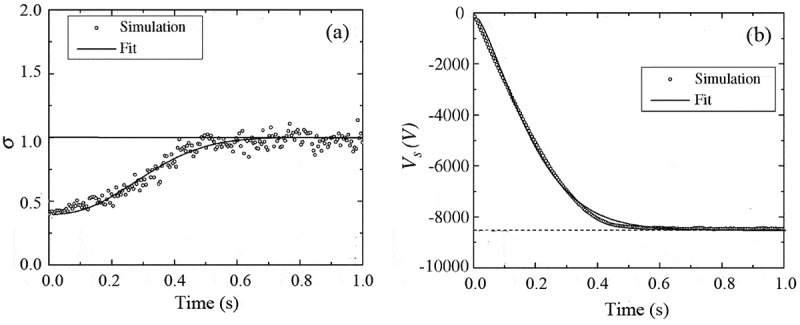
Simulated total electron yield (a) and surface potential (b) as a function of time for a semi-infinite SiO_2_ sample. In the simulation, the primary electron energy was taken as 10 keV, beam diameter as 200 μm, and trapping site density as 1 × 10^20^ cm^−3^, respectively. Adapted from Ref. [118] with permission from Elsevier

In contrast, negative charging effect requires a net accumulation of negative charges, and usually occurs in very low or relatively high primary electron energies where the total electron yield is lower than the unity. Figure 13(a) shows the simulated total electron yield as a function of time for a semi-infinite SiO_2_ sample when irradiated by a 10 keV primary electron beam [118], where it increases from a lower value up to the unity, resulting in the negative surface potential in Figure 13(b) due to the net accumulation of negative charges.

**Figure 14.**
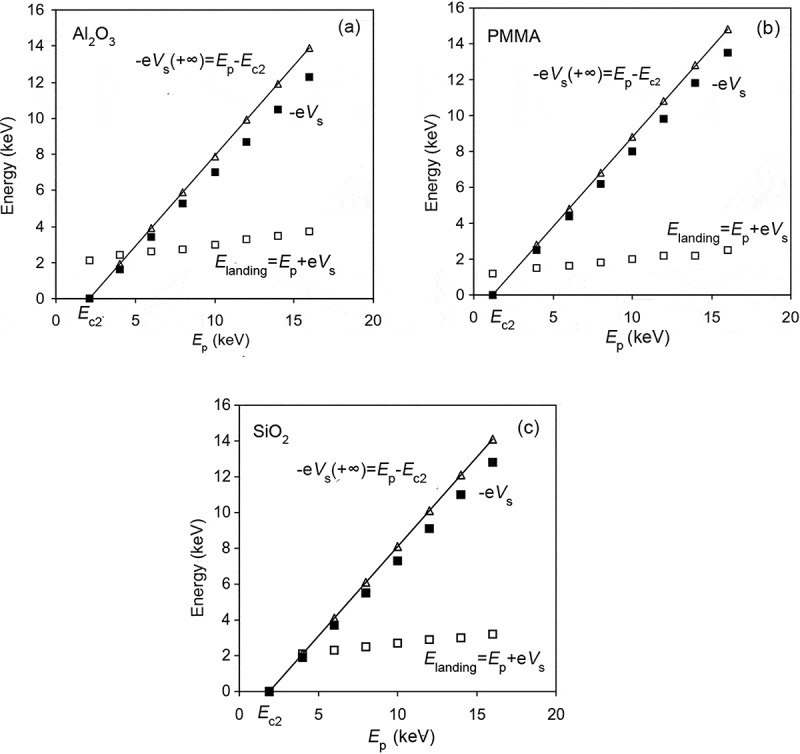
Experimentally measured deceleration magnitudes of primary electrons in the steady state of the charging effect as a function of the primary electron energy for (a) Al_2_O_3_; (b) PMMA; (c) SiO_2_. In (a)-(c), the solid squares represent the deceleration magnitudes of the primary electrons, $-eV_{s}$, which were determined from the shift of the secondary electron peak in the emitted electron energy spectrum; the open triangles represent the deceleration magnitudes estimated from the linear relation, $-eV_{s}=E_{p}-E_{c2}$; the open squares represent the final landing energy of primary electrons, $E_{landing}=E_{p}+eV_{s}$. Adapted from Ref. [28] with permission from Elsevier

It is of interest to see that the total electron yield in Figure 13(a) and the surface potential in Figure 13(b) vary with time in a synchronous manner, although their variation tendencies are opposite to each other. In fact, the self-modulation mechanism via which negative charging effect reaches the steady state can be inferred from Figure 13: the negative surface potential decelerates the primary electrons, giving rise to the reduction of the landing energy and the increase of the total electron yield, until the steady state is reached. From this point of view it can be inferred that negative charging effect will evolve along the reduced $\sigma_{r}$-curve in Figure 9(b) from a certain primary electron energy point $E_{p}$ at which σ<1 to the second critical primary electron energy $E_{c2}$ at which σ=1. This allows the surface potential in the steady state of negative charging effect, $V_{s}\left(+\infty \right)$, to be roughly deduced via $-eV_{s}\left(+\infty \right)=E_{p}-E_{c2}$, which has been experimentally verified for different insulating materials, as shown in Figure 14 [28]. Here the experimental deceleration magnitudes of primary electrons, $-eV_{s}\left(+\infty \right)$, were obtained by measuring the shift of the secondary electron peak in the emitted electron energy spectrum. And due to the linearity of the deceleration magnitude, the landing energy of primary electrons in the steady state of the charging effect is almost a constant, which is roughly equal to the second critical primary electron energy $E_{c2}$.

Kotera and Suga [119] have proposed a Monte Carlo model for calculating the trajectories of the primary electrons incident into insulating samples under a negative charging effect. Note that the involved negative charging effect is produced prior to the trajectory calculation by injecting a certain dose of electrons into the sample. In particular, the produced pre-charging is represented by the deposited charge distribution induced by one injected electron, which is averaged over the injection of 10^5^ electrons without taking the electric field into account. Then, the deposited charge distribution induced by a varying injected electron dose can be obtained by multiplying the distribution corresponding to one injected electron irradiation by the injected electron number. However, the obtained deposited charge distribution, which will be used for calculating the potential distribution, is assumed to maintain stationary, with its redistribution due to charge drift being omitted. Then, the calculation of the trajectory is done by using a single scattering model [120] where the electron elastic scattering is described by the screened Rutherford differential cross section formula while the electron energy loss between two adjacent elastic scatterings is calculated with the modified Bethe stopping power equation. Furthermore, the electron energy and transport direction are corrected for the electric field between adjacent elastic scatterings.

With this model, the authors simulated incidence of 100 electron trajectories of 20 keV into a thick polymethylmethacrylate (PMMA) wafer under the negative charging condition, which was produced in advance by irradiating the sample with a varying dose of 20 keV electrons. It was found that due to the repulsion by the electrons incident in advance into the sample, the distribution range of the primary electron trajectory is shrunk, and the more electrons incident into the sample in advance, the larger the shrinking extent of the trajectory-range will be.

### 4.3. Manifestations of charging effect

This section discusses the influence of the charging effect on experiments by reviewing the reported investigations in the fields of electron yield measurement, SEM imaging, EBL exposure and energy spectrum measurement.

#### 4.3.1. Electron yield measurements

The characteristics of the charging effect of a thick insulating foil described in Section 4.2 provide a guidance for measuring the electron yield of insulating materials. Experimentally, Blaise et al. [14] have measured the total electron yield of mica as a function of the beam dose at different primary electron energies. Features of their results obtained at the primary energy of 0.2 keV with the occurrence of positive charging effect and those at 6 keV with the occurrence of negative charging effect are the same as those exhibited in Figure 10(a) and 13(a), respectively, validating at least qualitatively the reasonability of the Monte Carlo simulations on the charging effect.

However, to measure the intrinsic electron yield of insulating materials, the charging effect should be minimized as much as possible. To achieve that, a pulsed primary electron beam with the pulse width ranging from ns to μs is usually employed, due to its advantage of allowing experimenters to control the beam dose conveniently. Usually, only few pulses are used to irradiate the sample, which is to keep the beam dose at a low level. Furthermore, the pulsed beam has mostly a defocused spot with a diameter ranging from µm to mm, further helping to minimize the charging effect. It is worth mentioning that the pulsed beam was also used in the experiment by Blaise et al. [14], whereas many more pulses were injected into the sample to deliberately produce an observable charging effect.

**Figure 15.**
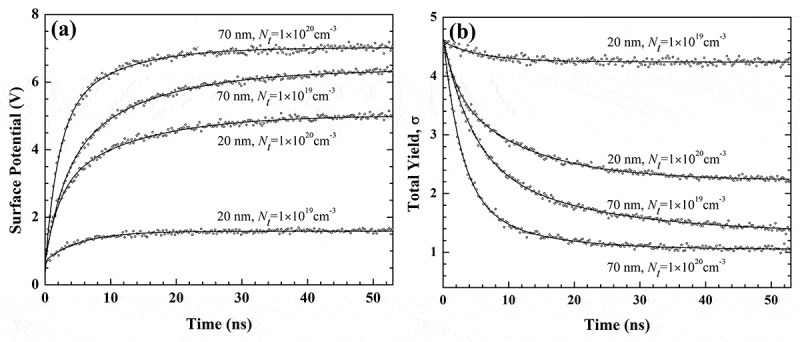
Simulated surface potential (a) and total electron yield (b) as a function of time for a SiO_2_/Au heterostructure. In the simulation, the primary electron energy was taken as 0.5 keV, beam diameter as 20 nm and beam current as 3 nA. Calculations were compared for samples with different film thicknesses (20 and 70 nm) and different trapping site densities (1 × 10^19^ and 1 × 10^20^ cm^−3^). Reprinted from Ref. [98] with permission from IOP publishing. © IOP Publishing. Reproduced with permission. All rights reserved

In the following we will focus on heterostructures as the sample in electron yield measurement. The first example is a SiO_2_ film grown on an Au substrate. Figure 15 shows the Monte Carlo simulated [98] positive surface potentials as functions of time for four cases of different film thicknesses and trapping site densities. The simulations were performed for an electron beam at primary energy of 0.5 keV and current intensity of 3 nA, and with beam diameter of 20 nm. The potential was calculated by using the multi-image charge method to incorporate the interactions of polarized charges on the SiO_2_-vacuum interface and those on the SiO_2_-Au interfaces. The simulation corrects the landing energy of the primary electrons at the sample surface due to the surface potential, and traces the transport trajectories of primary and secondary electrons in SiO_2_ according to the theoretical framework described in Section 2 until the electron energy is lowered down to the electron affinity, during which the electron trajectories in the sample are corrected for the electric field. In contrast, the electron transport in Au substrate is done in a similar manner to that in SiO_2_ but the electron inelastic scattering is described by FPA [60]. The simulation traces also the drift of the holes and the energy-exhausted electrons in SiO_2_ until they are trapped or recombined by the uniformly-set trapping sites according to the three occasions given in Section 3, allowing the acquisition of a charge distribution which is then used for calculating the potential distribution. Generally, the charging effect influences secondary electrons easily, but not primary and backscattered electrons. Thus, the simulation only corrects the landing energy of the incident primary electrons at the sample surface by considering the surface potential, but not change of their trajectories by the electric field in vacuum. Similar energy correction is also done for the emitting secondary and backscattered electrons at the sample surface by subtracting the surface potential from their kinetic energies; only the electrons with positive energies after correction are treated as emitted signals, while the rest ones are deposited at the surface sites where they are going to emit.

Impressively, the charging effect in Figure 15 is weakened with either decrease of film thickness or trapping site density, which can be accounted for by the combination of the following aspects. The first aspect is concerned with the primary electron leakage that the maximum penetration depth into SiO_2_ by 0.5 keV primary electrons in the charging-absent situation is about 20 nm according to the Monte Carlo simulation based on the theoretical framework given in Section 2. Obviously, a portion of primary electrons can penetrate the film into the substrate if the film is thinner than 20 nm, reducing the charge accumulation in the film. The second aspect is that the less trapping sites will reduce charge accumulation in the film. The third aspect is that the electrons in the substrate can be attracted upwards to the SiO_2_-Au interface by the net positive charges accumulated in the film. On the other hand, since the positive charging effect is weakened, the total electron yield in the steady state of the charging effect exceeds the unity, as can be seen in Figure 15(b).

It is worth noting that in this simulation a focused and continuous electron beam is considered, which is not in accord with the requirement on the electron beam for the absolute yield measurement of insulators [121]. However, even though the results presented in Figure 10(a) and 13(a) suffer the similar problem but they still agree qualitatively with the experiment by Blaise et al. [14], indicating that the use of a pulsed and defocused electron beam will not induce fundamental changes on the charging effect. Thus, the discussion presented above is still expected to be applicable to the electron yield measurement for this heterostructure.

**Figure 16.**
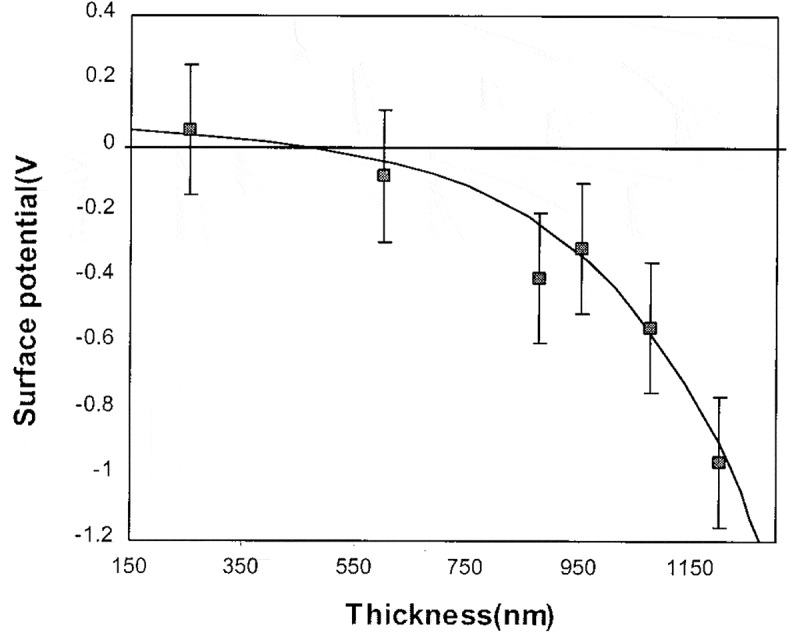
Surface potential for a UV5 resist coated on a Si substrate as a function of the resist thickness measured by a Kelvin probe micrometer. In the experiment, the primary electron energy was taken as 20 keV and the measurement was done after the incident primary electron dose delivered 10 µC cm^−2^. Reprinted with permission from Ref [122]. Copyright [1999], American Vacuum Society

The second heterostructure studied is an insulating resist layer coated on a Si substrate under the irradiation of an electron beam at a relatively high energy in order to guarantee that the induced charging effect is negative for the adequate thick layer. In contrast to the first heterostructure, the negative charging polarity for this heterostructure does not remain unchanged throughout the reduction of the film thickness, but instead can be transformed into the positive polarity. This is clearly seen in Figure 16 for the experimental measured surface potential for a UV5 resist layer coated on a Si substrate as a function of the resist-layer thickness by using a Kelvin probe micrometer [122]. The measurement was done after the irradiation on the resist layer by a 20 keV primary electron beam delivered the dose of 10 µC cm^−2^ in all the cases of different resist-layer thicknesses. As can be seen in Figure 16, the critical thickness is about 450 nm, beyond which negative charging effect occurs and below which positive charging effect occurs. This transformation, in principal, is due not only to primary electron leakage but also to the electron beam induced current (EBIC). The primary electron leakage can cause the loss of more electrons than holes for the resist layer, which, in conjunction with the enhancement of the primary electron leakage in the thinner resist layer case, weakens negative charging effect when the resist-layer thickness is decreased until positive charging effect appears. The EBIC, due to the push of the energy-exhausted electrons by the negative surface potential to the substrate, helps to minimize the negative charging effect of the resist layer. In addition to UV5, the similar surface potential as a function of the resist thickness to Figure 16 has also been observed for the PBS resist layer coated on a Si substrate [122].

**Figure 17.**
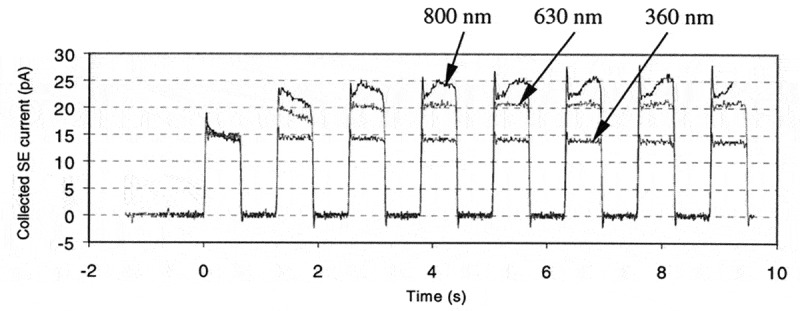
Measured secondary electron current emitted from a PMMA resist of different thicknesses, i.e. 360 nm, 630 nm and 800 nm, coated on a Si substrate. In the experiment, a 5 keV primary electron beam was deliberately controlled to irradiate the sample in certain time cycles with the duration of each cycle being 1.26 s. Reprinted with permission from Ref. [123] Copyright [2003], American Vacuum Society

This polarity-transformation charging effect on the electron yield can be seen from an experimental measurement (Figure 17) on the secondary electron current emitted from a PMMA resist layer coated on an Si substrate [123]. This experiment was done by using a SEM, in which a particularly developed planar detector was installed in the chamber to collect the emitted secondary electrons. However, a 5 keV primary electron beam was deliberately used to irradiate the sample in certain time cycles, in which non-zero secondary electron current was obtained. The maximum penetration depth into PMMA by 5 keV primary electrons is about 700 nm [123]. In the cases of 630 nm and 800 nm, the collected secondary electron current increases as a function of time and becomes approximately saturated in the third cycle, indicating that the charging effect involved in these two cases are both negative. Experimentally, the planar detector was installed directly above the sample, so it can collect the secondary electrons emitted in a certain solid angle symmetrical to the normal of the resist surface, but not those secondary electrons flying nearly parallel to the resist-layer surface. The point is that once the resist layer is negatively charged, the emitted secondary electrons, especially those ones flying nearly parallel to the resist-layer surface, can be repelled upwards, giving rise to the enhanced secondary electrons in the detection solid angles and to the increase of the detected secondary electron current. Furthermore, the saturated secondary electron current in the 800 nm case is greater than that in the 630 nm case, from which it can be inferred that the charging effect in the 800 nm case is stronger than in the 630 nm case. However, the secondary electron current in the 360 nm case is the smallest and becomes saturated in the first time cycle, indicating that the charging effect involved in this case is positive.

#### 4.3.2. SEM imaging

In Section 4.3.1 it is described that the charging effect can alter electron yield to a big extent, which accordingly affects SEM imaging, as can be seen from the following examples.

**Figure 18.**
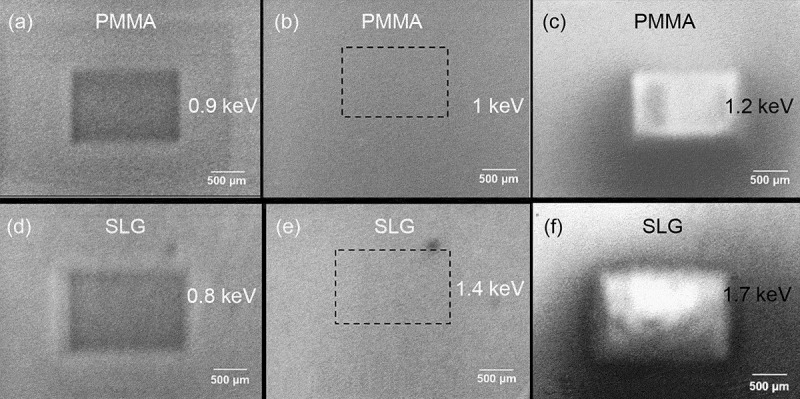
Experimental secondary electron images of semi-infinite PMMA and SLG samples obtained at different primary energies: (a) 0.9 keV, (b) 1 keV and (c) 1.2 keV for PMMA; (d) 0.8 keV, (e) 1.4 keV and (f) 1.7 keV for SLG. Adapted from Ref. [28] with permission from Elsevier

The first example [28] is an experimental imaging of the planar surfaces of the semi-infinite PMMA and soda-lime glass (SLG) samples at different primary electron energies by following a three-step imaging procedure according to Joy and Joy [124]. The first step is to scan a surface region at a certain magnification, the second step to increase the magnification and scan a central rectangular part of the region just scanned previously, and the third step to restore the magnification and scan the region in the first step again. The obtained secondary electron images are shown in Figure 18, where the dark rectangular regions in Figure 18(a,d), the bright rectangular regions in Figure 18(c,f), and the rectangular region defined by a dotted boundary in Figure 18(b,e) are just the central rectangular part scanned in the second step. In this example, contrast should not be exhibited in all images if everything is normal according to the conventional SEM imaging principle. However, the experiment showed remarkable contrasts in some images of Figure 18, implying a unconventional physical factor for the contrast. This factor can be associated with the charging effect due to the insulation nature of the samples. To be more specific, Figure 18(a,d) show the occurrence of positive charging and the image darkening in the central rectangular region is naturally due to the SEE impediment; Figure 18(c,f) indicates negative charging effect, where the image brightening is due not only to the SEE enhancement induced by primary electron deceleration but also to the collection enhancement of secondary electrons since those ones flying nearly parallel to the sample surface could be impelled upwards. In contrast, Figure 18(b,e) are normal images since there is almost no contrast shown therein, implying the absence of the charging effect. Furthermore, for the PMMA sample, the primary electron energy in Figure 18(c) is greater than that in Figure 18(a) and that in Figure 18(b) is in the moderate amplitude, so does the SLG sample; this is in accord with the discussions in Sections 4.1 and 4.2 on the primary electron energy range in which different charging effects occur. This darkening and brightening in the secondary electron image can be regarded as typical features for occurrence of positive and negative charging effect in SEM imaging, respectively. This three-step imaging procedure is an effective approach for ascertaining the second critical primary electron energy.

**Figure 19.**
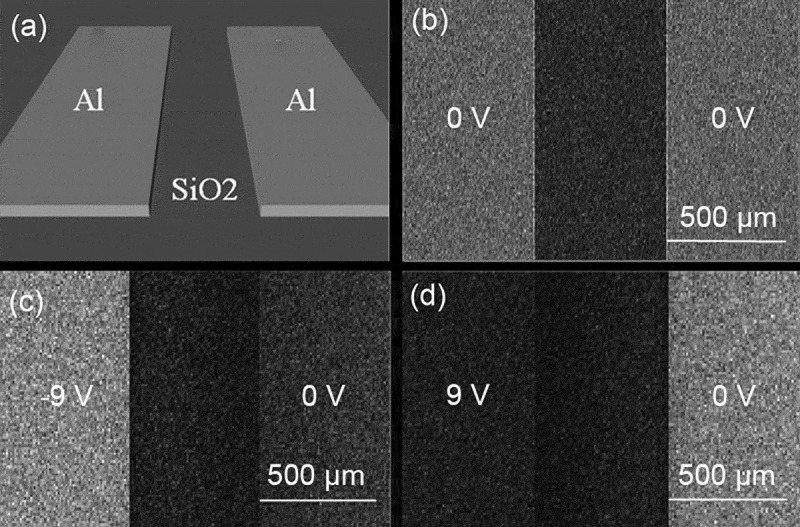
Simulated secondary electron images for two Al electrodes placed on a SiO_2_ substrate at the primary electron energy of 1 keV. The configuration of the simulated sample is shown in (a). In the simulation, a voltage bias was applied to the left electrode, while the right electrode was always grounded. The voltage bias applied to the left electrode is: (b) 0 V; (c) −9 V; (d) 9 V. Adapted with permission from Ref [125]. Copyright [2010], American Vacuum Society

Darkening and brightening in secondary electron images have also been observed in the second example of a simulated SEM imaging at the primary electron energy of 1 keV by Babin et al. using a Monte Carlo method [125], as shown in Figure 19. Their sample consists of a SiO_2_ substrate on which two Al electrodes are placed parallel to each other, as can be seen in Figure 19(a). In the model, the electron transport is traced in the sample by handing their encountered elastic and inelastic scatterings using the CHARIOT software [126,127] until the electron energy decreases to the work function. The sample geometry is constructed with this software as of a multi-layer structure, whereas each layer has a relatively simple geometry. By the simulation of the drift and diffusion of the electrons and holes and by taking their trapping and recombination into account, the charge distribution can be obtained. The electric field generated by this charge distribution is then calculated for correcting in vacuum the incident trajectories of primary electrons and the emitted trajectories of secondary electrons, in which the divided grids are non-uniform and are distributed densely with the smaller sizes close to the sample but with the larger sizes far from the sample. In calculating the potential distribution, the metallic sub-regions G are taken as equipotential bodies, with their potentials given by

(39) $$\phi_{G_{i}}=\Phi_{i},\;\;i=1,2\cdots N,$$

where Φ is the potential value and N is the number of the metallic sub-regions contained in the sample. The determination of Φ has to resort to the Gauss’s theorem,

(40) $$\int_{{\;}_{G_{j}}}\varepsilon {\left(\nabla \phi \right)}_{n}ds_{G_{j}}=-q_{j}/\varepsilon_{0},\;\;j=1,2\cdots N,$$

where ε is the dielectric constant of the matrix where the metallic region is contained and q the charge quantity accumulated in the metallic sub-region; the integral runs over the boundary of the metallic sub-regions, with ${\left(\nabla \phi \right)}_{n}$ being the electric field close to the boundary and perpendicular to it. Equations (39) and (40) are the boundary conditions that have to be satisfied in the calculation of the potential distribution for the samples containing metallic sub-regions.

After applying a varying voltage bias of 0 V, −9 V and 9 V to the left electrode and a constant 0 V to the right electrode, the secondary electron images shown in Figure 19(b–d) were obtained, respectively, where the variation of the contrast with the voltage bias can be seen clearly. Essentially, the applied positive and negative voltage bias impact the SEE in the manner similar to the previously discussed positive and negative charging effect, respectively, and the 0 V case corresponds to the absence of the charging effect. It thus comes in Figure 19 that in contrast to the right electrode, the left electrode becomes darker if a positive voltage bias is applied and brighter if a negative voltage bias is applied; the situations faced by these two electrodes will be the same if a 0 V voltage bias is applied to the left electrode, leading naturally to the absence of the contrast between them. The agreement of this simulation with the experimental result has been shown in Ref [125].

However, the sample similar to that investigated by Babin et al. [125] can also be charged even when the upper metallic structure is floating, i.e. it is not connected to any voltage bias, as can be found from the Monte Carlo simulation by Villarrubia et al. [128]. Their simulation focused on the situation that the sample, constituted by a SiO_2_ substrate and a floating metallic photomask with a trapezoidal cross-section placed on the substrate, was irradiated by a scanning electron beam. In particular, the photomask itself can have accumulated charges if the number of electrons entering into it does not equal to those leaving it, especially considering the substrate is insulating such that the charges in the photomask cannot flow to the ground or be recombined by the charges in the ground. Meanwhile, the region selected for irradiation in the simulation included a part of the substrate surface, causing the substrate charging. The simulation was based on a Monte Carlo simulation package, JMONSEL [129,130]; it uses the so-called constructive solid geometry (CSG) method to construct the sample geometry via assembling a certain number of primitive geometries, including spheres, cylinders, and multi-plane geometries, in an appropriate manner and one can gain more insights into this method by referring to its applications in other Monte Carlo simulations [57,131]. In this model, the space is meshed inside and outside the sample with numerous tetrahedra of varying sizes, which are distributed densely close to the sample and sparsely far from the sample to speed up the calculation, and this mesh is integrated into JMONSEL by allowing the used tetrahedra to be sub-regions in the standard CSG representation. In the simulation, by saving in the tetrahedra the positions where holes are generated and those where the electron trajectories are terminated, a charge distribution can be obtained and the simulation does not take the further drift of the charges into account. Thereafter, it starts to use FEA to calculate electric field for tracing and correcting trajectories of the emitted secondary electrons in vacuum. In calculation of the electric field, the boundary conditions chosen for the metallic photomask are the same as Equations (39) and (40). It was found with this model that the sample was positively charged at the primary electron energy of 1 keV in the situation mentioned above, indicated by the obtained positive potential on the photomask surface. In FEA there is a trade-off, with speed and economy of memory favoring a coarse mesh and accuracy favoring a fine one, which can be optimized through the use of adaptive mesh refinement [132]. The goal of adaptive mesh refinement is to automatically generate a mesh that is fine in the regions where small elements are needed to accurately resolve the solution, and coarse in regions where large elements suffice.

**Figure 20.**
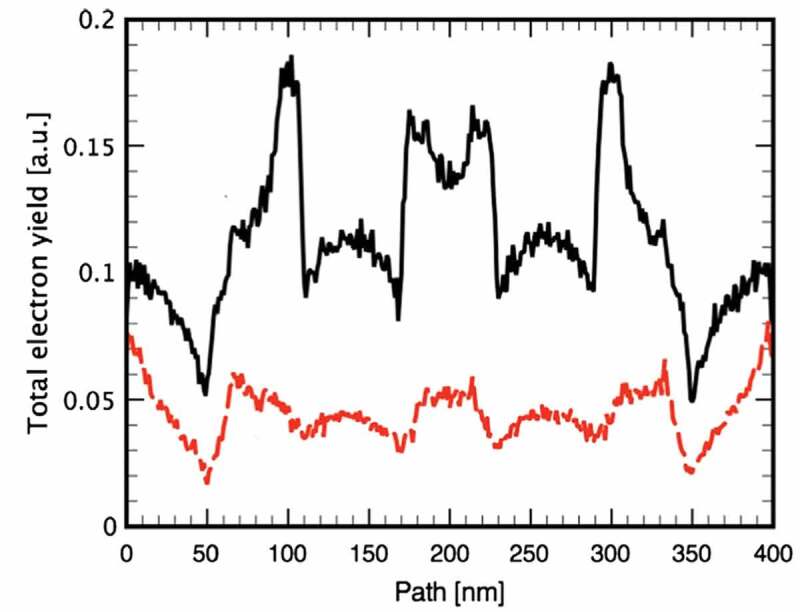
Comparison of the total electron yield profile obtained in a line scanning at the primary electron energy of 1 keV without (black curve) and with (red curve) considering the electric field. Adapted from Ref [133]. with permission from Elsevier

Ciappa et al. have also proposed a Monte Carlo model for simulating the charging effect in 3D structures [133]. Their modeling for mesh the space inside and outside the sample is similar to that by Villarrubia et al. [128], i.e. using tetrahedra as the mesh unit with varying densities at different positions. The computer package IES [134] is used to trace the electron transport. Charge distribution is obtained by saving the positions of holes where they are generated and those of electrons where their transport is terminated, without taking their drift into account, for the calculation of the electric field within the TCAD environment. The calculated electric field is passed to IES for correcting the electron trajectories inside and outside the sample, giving rise to a remarkable increase of the simulation time to 190 ms for a single primary electron irradiation from that of 20 ms without the correction.

Figure 20 compares the electron yield profiles for three trapezoidal SiO_2_ lines grown in parallel on a Si substrate, which are obtained in a line scanning across the SiO_2_ lines with and without taking the electric field into account at the primary energy of 1 keV. In particular, the electric field is induced by a pre-scanning at the primary electron energy of 1.5 keV along the line. It can be seen that the electric field causes an overall reduction of the total electron yield, which is due to the attraction of a portion of secondary electrons back to the sample surface and indicates typically that the charging effect involved in this case is positive.

**Figure 21.**
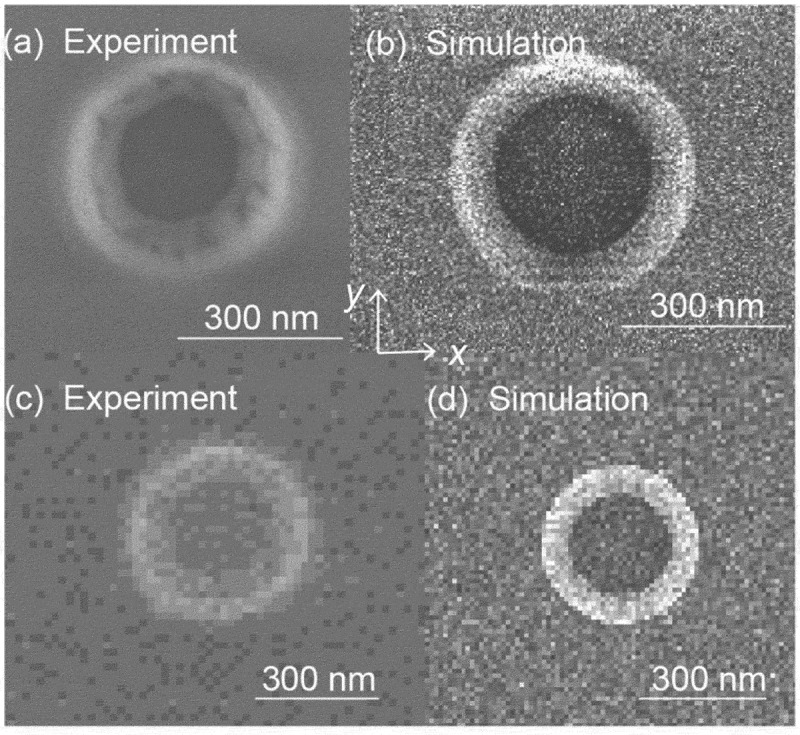
Comparison of the secondary electron image of an etched SiO_2_: (a) experiment, high magnification; (b) simulation, high magnification; (c) experiment, low magnification; (d) simulation, low magnification. Adapted from Ref. [135]. with permission from John Wiley & Sons, Inc

However, positive charging effect can sometimes improve SEE for very special structures, as can be found from the following SEM imaging by Grella et al. [135,136]. Their simulation model combines a ray tracing technique and a Monte Carlo method, which are used for tracing electron transport in vacuum and in the sample. The adopted Monte Carlo method is also a single scattering model including merely the electron elastic scattering described by the Mott’s cross section, while the energy loss of the transport electrons between adjacent elastic scattering events is evaluated by Bethe stopping power formula. The model considers the accumulation in the sample of positive charges due to SEE and that of negative charges due to energy exhaustion of electrons. By solving the Poisson equation, the electric field generated by those accumulated charges is obtained, which will be used for correcting the primary and secondary electron trajectories in vacuum. In using this model to simulate the charging effect for a 3D structure with a micrometer size at the primary electron energy of 0.6 keV, the calculation time was in the order of hours on a personal computer with a dual 3.06 GHz processor.

This model was then applied to simulate SEM imaging for an etched circular in SiO_2_ via at different magnifications [135]. The scanning was performed at first along a line parallel to the *x*-axis, after which the scanning along the next line, which was also parallel to the *x*-axis but was moved a certain distance along the *y*-axis, was performed, and so forth. The simulated secondary electron images agree with the experiment, as shown in Figure 21. However, the bottom of the etched via is slightly brighter at the low magnification (Figure 21(c,d)) than at the high magnification (Figure 21(a,b)). The reason for this observation was considered to be that the potential at the top became more positive in the low-magnification situation, such that the secondary electrons originated from the via bottom could be attracted upwards for emitting into vacuum. Furthermore, the images are slightly stretched along the *y*-direction, which is more obvious for the high-magnification situation, and it was attributed to the influence of the local electric field on electron trajectories in vacuum.

Though the Monte Carlo models mentioned above can be used to investigate the charging effect for 3D structures, they either cannot construct very complex structures or do not take the drift of the holes and the energy-exhausted electrons into account. These problems are properly handled by another Monte Carlo model [98], CTMC-CHARG [114], where the 3D structures are constructed by using the finite triangle mesh (FTM) method and the trapping and recombination of the holes and the energy-exhausted electrons have been taken into account. FTM relies on placing a group of planar triangles tightly on the surface of the aimed structure, where the triangles are allowed to have different geometries and sizes, enabling the construction of very complex 3D structures [137–141]. Approaches used for describing electron transport, charge drift and SEE are the same as that for Figure 15, while the potential distribution is calculated by using the self-consistent method.

**Figure 22.**
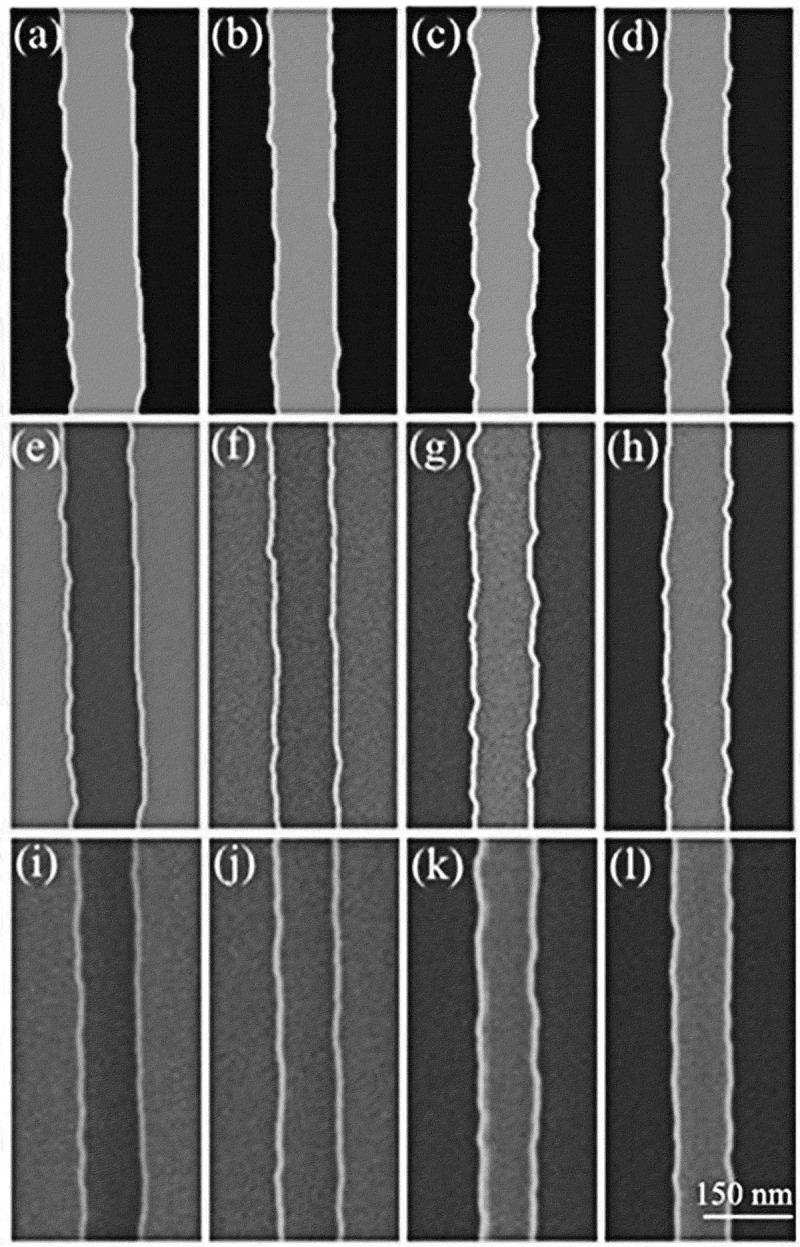
SEM images of trapezoidal SiO_2_ line structure grown on a Si substrate. (a)-(d) are the simulated images without taking charging into account; (e)-(h) are the simulated images by taking charging into account; (i)-(l) are the experimental images. From the left-most column to the right-most column, the primary energies are 0.2, 0.4, 0.8, and 2 keV. In the simulation, the trapping site densities in the SiO_2_ trapezoid and on the SiO_2_/Si interface were taken as 1 × 10^19^ cm^−3^ and 1 × 10^14^ cm^−2^, respectively. Reprinted from Ref [98]. with permission from IOP publishing. © IOP Publishing. Reproduced with permission. All rights reserved

To test their model, the authors applied it at first to investigate the charging effect exhibited in the SEM imaging of a SiO_2_ trapezoidal line grown on a Si substrate, which is a fundamental structure in the manufacture of integrated circuits and is usually characterized by CD-SEM for confirmation of critical dimension (CD). A contrast reversal phenomenon has been observed in experimental secondary electron images for this structure [142]. Figure 22 shows the experimental secondary electron images and the simulated ones with and without taking the charging effect into account [98]. Ideally, the normal experimental images should be like Figure 22(a–d), in which the SiO_2_ line is always brighter than the Si substrate, since the secondary electron yield of SiO_2_ is higher than that of Si in the involved primary electron energy range. However, the contrast reversal was observed in the experiment that the SiO_2_ line changes from darker to brighter than the Si substrate as the primary electron energy is increased from 0.2 keV to 2 keV, as shown in Figure 22(i–l), which was attributed to the charging effect [142]. The contrast of the simulated images taking the charging effect into account in Figure 22(e–h) agrees with that of experimental ones, indicating that this phenomenon is indeed caused by the charging effect. Furthermore, the charging effect in the cases with different primary electron energies was confirmed to be all positive by their positive spatial potential distribution obtained in this simulation. Nevertheless, the magnitude of their positive spatial potential is different with each other and approximately decreases with the increasing primary electron energy, resulting in the decrease of the SEE impediment suffered by the upper trapezoidal line and in the appearance of the contrast reversal phenomenon.

**Figure 23.**
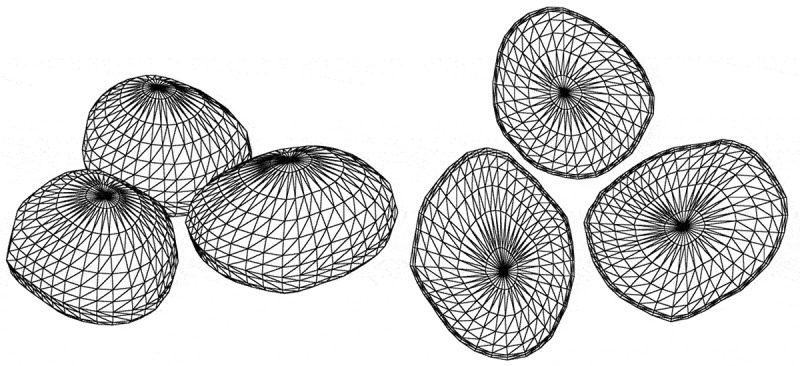
Geometry of irregular SiO_2_ nanoparticles constructed using the FTM method. Reprinted from Ref [98]. with permission from IOP publishing. © IOP Publishing. Reproduced with permission. All rights reserved

**Figure 24.**
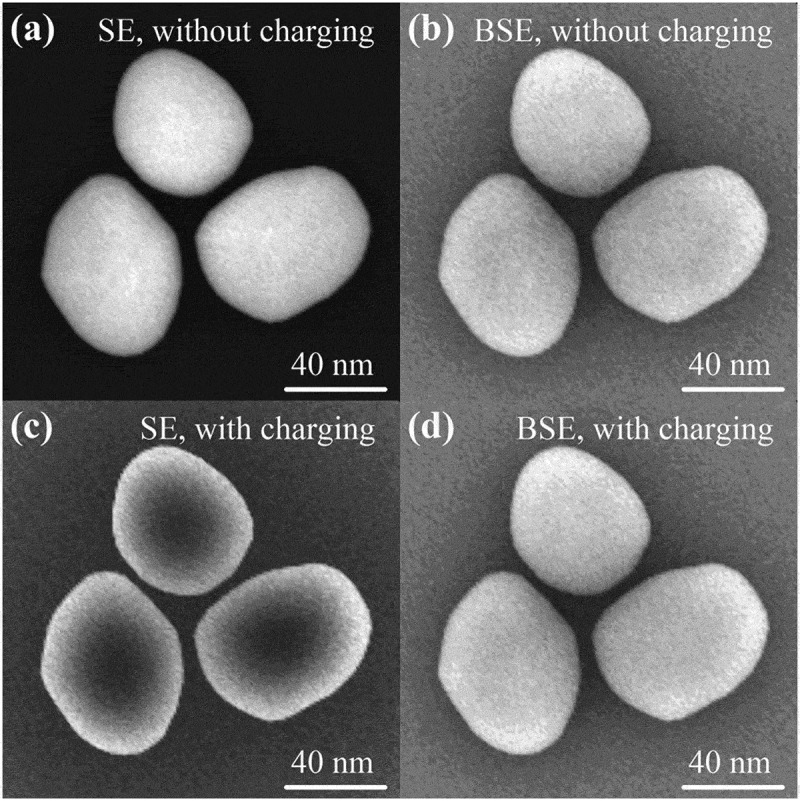
Simulated secondary electron (SE) image and backscattered electron (BSE) image for irregularly shaped SiO_2_ nanoparticles on a Si substrate with and without taking the charging effect into account. (a) SE image without charging; (b) BSE image without charging; (c) SE image with charging; (d) BSE image with charging. In the simulation, the primary energy is 1 keV, and the trapping site densities in the nanoparticle and on the SiO_2_/Si interface are 1 × 10^19^ cm^−3^ and 1 × 10^14^ cm^−2^, respectively. Reprinted from Ref [98]. with permission from IOP publishing. © IOP Publishing. Reproduced with permission. All rights reserved

Besides, the model was applied to simulate the SEM image for another structure, i.e. irregularly shaped SiO_2_ nanoparticles on a Si substrate, which is more complex from the consideration of geometry construction with FTM (Figure 23). Figure 24 compares the simulated secondary and backscattered electron images without and with taking the charging effect into account at the primary energy of 1 keV. Due to the enhanced emission of secondary electrons and backscattered electrons from the central region of a nanoparticle by its size and geometry, the central region is brighter than the outer region if the charging effect is not involved, as shown in Figure 24(a,b). However, the variation in the contrast due to the charging effect is remarkable for the secondary electron image in Figure 24(c), where the central region becomes darker than the outer region, but the charging effect is negligible for the backscattered electron image in Figure 24(d). Similarly, the spatial potential distribution obtained in this simulation tells also the occurrence of positive charging effect, whereas its magnitude is larger and can reach roughly 20 V due to the special geometry. In particular, the potential reaches its maximum in the central region of the nanoparticle, giving rise to the greater SEE impediment there and to the above mentioned contrast change in the secondary electron image. Though the potential exceeds that for the semi-infinite samples, it is still not adequate to induce remarkable change in the backscattered electron image, considering the energies of backscattered electrons are usually high [143–145].

**Figure 25.**
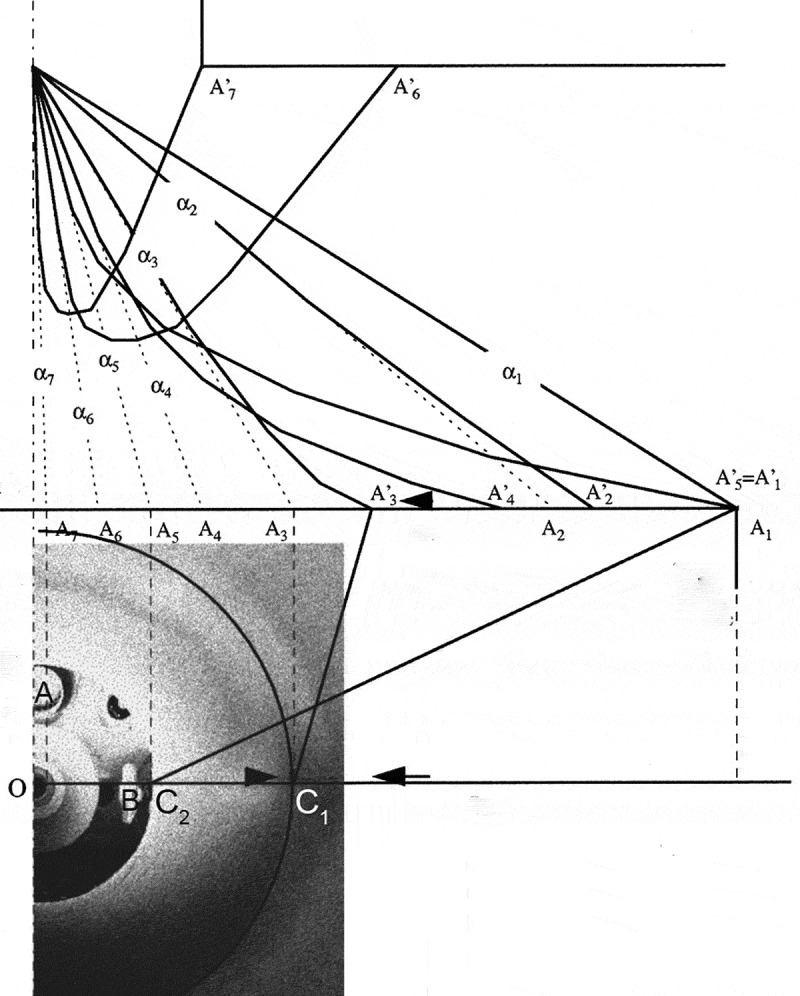
Experimental secondary electron image with mirror imaging at the primary energy of 1 keV. This image was also obtained via the conventional pixel-by-pixel imaging procedure, prior to which a certain amount of negative charges was injected into the center of this image as a point charge. Several annular bands are manifested in the inner part of this image. Regions A and B correspond to the secondary electron detector and the backscattered electron detector, respectively. The cause of this special imaging is shown schematically, in which α denotes the initial incidence angle of the primary electron beam relative to the sample surface normal, A the point on which the primary electron should irradiate, and A’ the point on which the primary electron actually irradiate. Adapted from Ref [146]., with the permission of AIP Publishing

The charging effects discussed above are kind of gentle, whereas in some cases the charging effect can impact SEM imaging quite severely. Figure 25 shows a typical secondary electron image obtained at the primary energy of 1 keV with the occurrence of mirror imaging and the schematic mechanism for this phenomenon [146]. It has to be noted that this mirror imaging followed also the conventional pixel-by-pixel imaging procedure, but prior to that a certain amount of negative charges was injected, as a point charge, into the sample. The point is that several annular bands are manifested in the inner part of the image. Careful analysis by the authors shows that the bright regions A and B in the image correspond, respectively, to the secondary electron detector and the backscattered electron detector, implying that the scene in the inner part of the image is belonging to the SEM chamber. For its cause, as can be seen from the schematic diagram, the primary electron beam with an initial incidence angle of α relative to the sample surface normal should irradiate on point A but actually irradiates on A’ due to its deflection by the negative charges injected to point O in advance. Depending on α, the primary electron beam can be so severely deflected that it can collide the chamber surface or the regions on the sample surface far from point O. With this picture, the scene in the image from C_1_ to C_2_ shows the sample morphology from A’_3_ to A’_5_, while that from C_2_ to O shows the inner scene of the SEM chamber. Since the fundamental cause of this special imaging is the deflection of the primary electron beam, which is analogous to the deflection of a beam of light by a mirror, it can thus be named as mirror imaging.

#### 4.3.3. EBL exposure

In addition to mirror imaging, the primary electron deflection can be encountered in common negative charging effect induced by primary electron irradiation in EBL exposure, but with a smaller deflection magnitude than the mirror imaging.

EBL does not rely on detecting any emitted electron signals but instead on accurately exposing the resist using the primary electron beam. However, the resist itself or the substrate on which it is placed may be insulating, naturally leading to the charging effect. Similarly, the occurrence of negative charging effect is quite possible for EBL at a high primary energy, in which the primary electron beam can be deflected since the negative surface potential is comparable in magnitude to the primary energy. Due to the beam deflection, the actual exposure position will be displaced from the prescribed pixel, which is an important problem as it lowers down the EBL quality. Arat et al. [147] have investigated this problem quantitatively by using a Monte Carlo method; for the sake of simplicity, the following discussion is focused on their simulation of exposing a single SiO_2_ substrate at the primary energy of 50 keV with the resist being assumed not to exist.

The simulation traces the electron transport step by step by individually handing the elastic and inelastic scatterings. The elastic cross section in the electron energy range of greater than 0.2 keV is calculated by using the ELSEPA package [76] and that in the range of lower than 0.1 keV is taken as the electron-acoustic phonon scattering cross-section; the interpolation between these two ranges gives that in the range of 0.1–0.2 keV. For the electron inelastic scattering, it is described by a dielectric functional approach, in which the Ashley’s model [148] with the refinements suggested by Kieft and Bosch [149] is adopted. Each electron is traced until its energy decreases to 10 eV. The trace-terminated electrons, however, can in fact still transport in reality until their energy is exhausted. As mentioned previously, the energy-exhausted electrons and the holes can drift in the electric field until they are trapped or recombined. All of these processes will cause a redistribution for the trace-terminated electrons and the holes, which is taken into account by describing their motion with an analytical position- and time-dependent electron beam induced conductivity into the simulation, as done by Koteral et al. [150]. Meanwhile, the breakdown of the material is considered in the way that the region is regarded as conducting if the electric field therein exceeds the threshold value chosen empirically.

**Figure 26.**
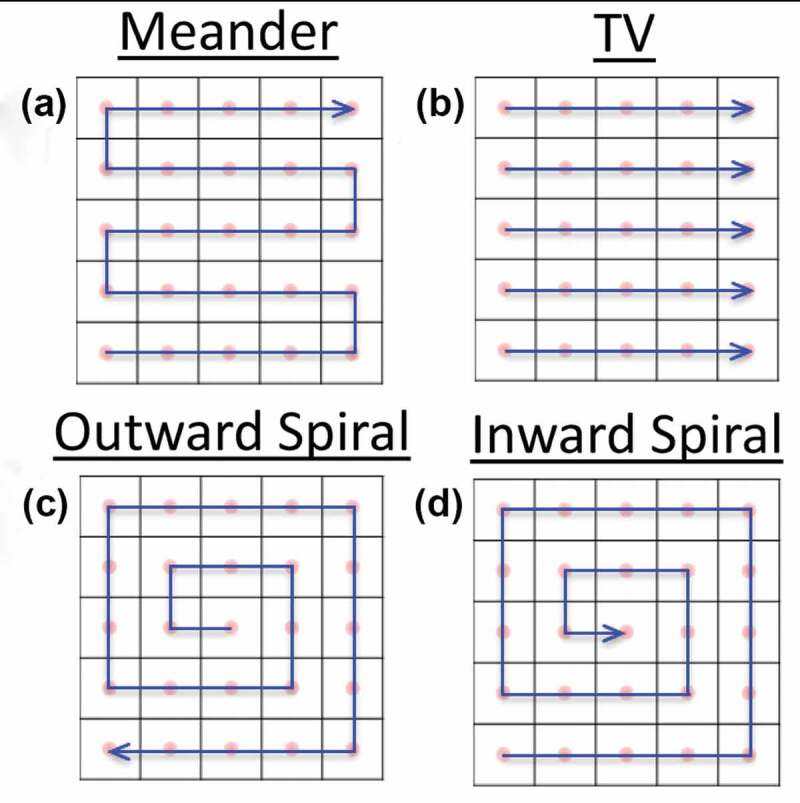
Four scanning modes considered in the simulation: (a) meander; (b) TV; (c) outward spiral; (d) inward spiral. In (a)-(d), the red dot array denotes the pattern to be exposed and the blue arrows denote the scanning order of the dots. Adapted with permission from Ref [147]. Copyright [2019], American Vacuum Society

**Figure 27.**
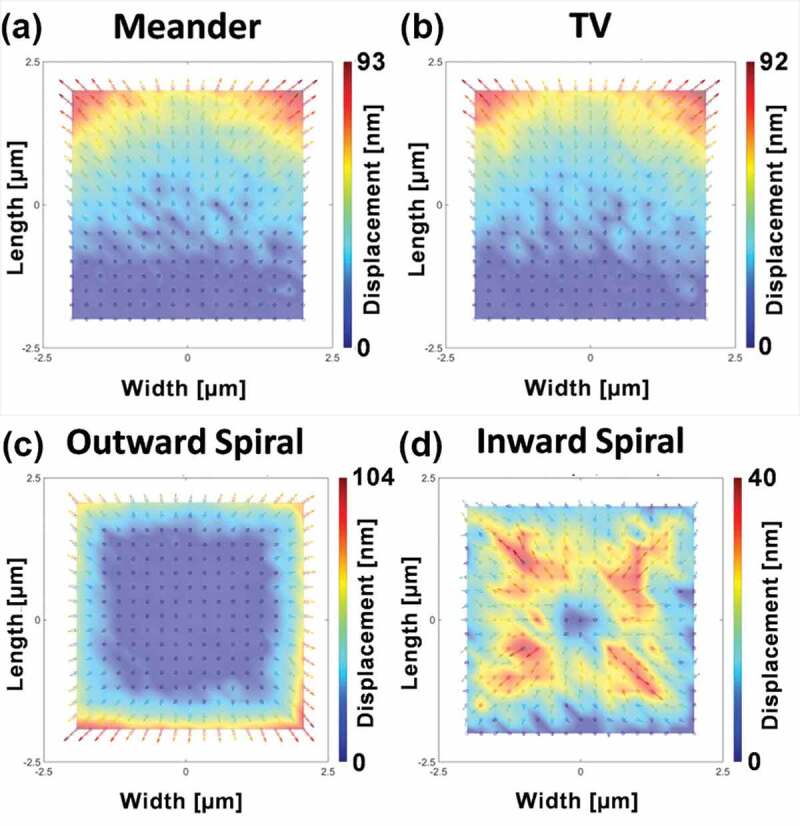
Simulated displacement of the primary electron beam when exposing a single SiO_2_ substrate under the scanning modes shown in Figure 26. In (a)-(d), the pattern under exposure is a 17□17 dot array with a 250 nm pitch, and the exposure dose is 128 µC cm^−2^. The arrows denote the displacement of the primary electron beam, with their directions denoting the displacement directions and their lengths denoting the displacement magnitudes. The displacement magnitudes can also be known from the color map in each figure. Adapted with permission from Ref [147]. Copyright [2019], American Vacuum Society

Four scanning modes were considered in the simulation, i.e. meander, TV, outward spiral and inward spiral, which are schematically shown in Figure 26. With these four scanning modes, the simulated displacements for the 17□17 dot array with a 250 nm pitch when the exposure dose reaches 128 µC cm^−2^ are shown in Figure 27. It can be seen that the displacements under the meander, TV and outward spiral modes have similar magnitudes ranging from 0 nm to about 100 nm, and are about twice of that under the inward spiral mode. As the size of the manufactured device is continuously decreasing, the pattern displacement with such magnitudes can be fatal for EBL. Though features of the displacements under different scanning modes are quite different, there still exists a common point among them, that is, the displacement at a point exposed at a later time is greater. This is due to that the charging effect becomes stronger as more and more primary electrons are incident into the substrate, naturally leading to a greater displacement of the primary electron beam. In addition, the primary energy is 50 keV, at which the charges accumulated in the substrate are dominated by electrons. This, in conjunction with the scanning order of the dots, determines the direction to which the primary electron beam is deflected; for example, in the meander mode, the scanning starts from the bottom to the top, so when exposing the upper pixels the primary electron beam is deflected upwards by the electrons accumulated in the lower regions.

#### 4.3.4. Energy spectrum measurement

Under charging condition the energy spectrum of the emitted electrons is timely changing, as can be found from the simulated shift of the secondary electron peak in the energy spectrum by Li et al. [151] by using a Monte Carlo method. In this model, the electron transport is traced according to the theoretical framework detailed in Section 2. However, in simulating the charging effect, an analytical Gaussian function is used to describe the energy distribution of the trapping sites that are uniformly set in the sample, and the electric-field-dependent charge drift velocity and trapping cross section are used to describe the charge drift and trapping, respectively. Furthermore, the detrapping of the trapped charges when the local electric field is adequately intense is also taken into account.

On the other hand, the configuration on which this simulation will be focused in the following is the irradiation of a semi-infinite sample by a normally incident focused electron beam, such that the involved charging condition is symmetric relative to the beam incidence direction, in which the electric field in the region surrounding the beam incidence direction does not have the lateral component. With this consideration, the simulation does not correct the trajectory for primary electrons in vacuum, but corrects their energy when reaching the sample surface. Furthermore, the simulation corrects also the trajectory and energy of the primary and secondary electrons inside the sample, and as for the emitted secondary electrons, their energies are corrected at the sample surface but not their trajectories in vacuum. The approach is the same as that in Ref [98].

**Figure 28.**
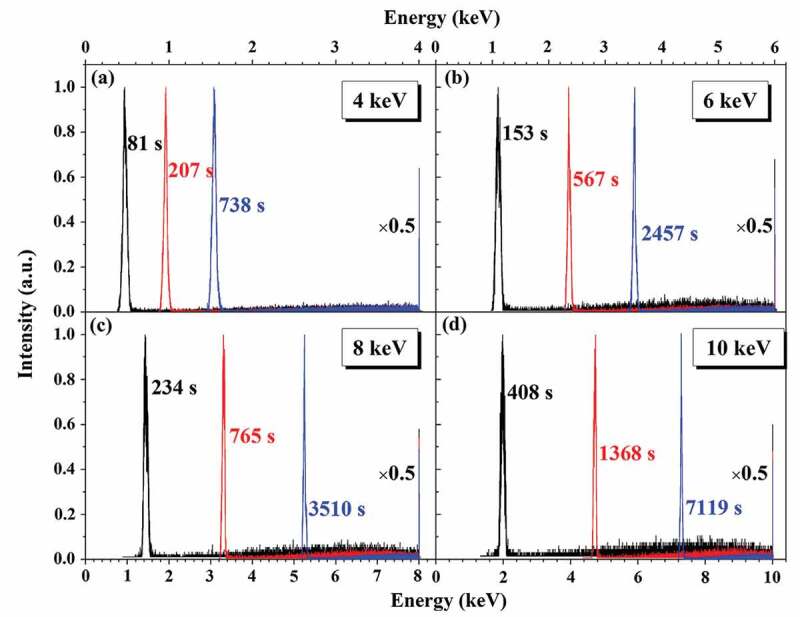
Simulated energy spectrum of all the electrons emitted at different time instants from a semi-infinite SiO_2_ sample due to the irradiation by a primary electron beam with energy: (a) 4 keV; (b) 6 keV; (c) 8 keV; (d) 10 keV. There are two sharp peaks in each energy spectrum, the left one being the secondary electron peak and the right one being the elastic peak. Reprinted from Ref [151]. with permission from Elsevier

Figure 28 shows the simulated energy spectra of all the electrons emitted at different time instants from a semi-infinite bulk SiO_2_ sample due to the irradiation by a primary electron beam with different energies. Remarkably, in each energy spectrum, the secondary electron peak (the left sharp peak) continues to shift to the higher energy side with time, while the elastic peak (the right sharp peak) maintains at a stable energy position. This peak shift is directly due to the acceleration of the emitted secondary electrons, implying the occurrence of negative charging effect. Furthermore, as mentioned previously, the magnitude of negative charging effect becomes increasing with the increasing primary electron energy, which is manifested in Figure 28 as the increasing peak-shift magnitude from Figure 28(a–d). In contrast, the variation of the elastically reflected electron energy due to the charging effect is proportional to the difference of the potential at the electron gun and that at the detector, and is equal to zero since these two points are far from the sample surface; thus, the elastic peak has a stable energy position.

In addition, this peak-shift phenomenon is applicable to the measurement of the surface potential, since the peak-shift magnitude is measurable in experiment and is just equal to that of the surface potential. And compared to the surface potential measurement using the experimental instruments, such as the Kelvin probe micrometer [122], the peak-shift method is more convenient in investigating the dynamic evolution of the surface potential from the very beginning of the primary electron irradiation to the steady state by collecting numerous energy spectra in this time period. Note that the deceleration magnitudes of primary electrons in Figure 14 [28], which are essentially the surface potentials, were just measured by using this peak-shift method.

Positive charging effect, on the other hand, can also impact the electron energy spectrum; but on the contrary the secondary electron peak shifts to the lower energy side [151], due to the deceleration of secondary electrons induced by the positive surface potential. Correspondingly, the maximum of the secondary electron peak is shifted into the negative energy range in the simulation. For the measurement of surface potential, a positive potential being equal to or greater than the surface potential must be applied in front of detector entrance in order to collect secondary electrons.

#### 4.3.5. Trapped charge distribution

It has to be clear that the charging effect is originated from the charge trapping in the insulating sample, so the knowledge of the spatial distribution of the trapped charges is benefit for the understanding of the charging effect. A theoretical model widely used for describing the trapped charge distribution in the sample induced by electron beam irradiation is the DDM [51], as has been mentioned in the introduction. However, DDM is actually an assumption and does not involve comprehensively the sophisticated electron-solid interactions.

**Figure 29.**
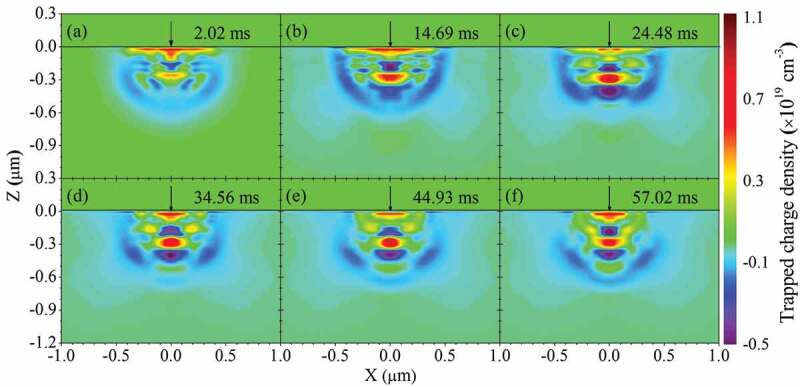
Simulated trapped charge distribution in a semi-infinite SiO_2_ sample due to the irradiation by a primary electron beam. (a)-(f) are distributions obtained at different time instants: (a) 2.02 ms; (b) 14.69 ms; (c) 24.48 ms; (d) 34.56 ms; (e) 44.93 ms; (f) 57.02 ms. In the simulation, the primary energy, the current intensity, the beam diameter and the trapping site density were taken as 5 keV, 0.1 nA, 0.1 µm and 2□10^19^ cm^−3^, respectively. Reprinted from Ref [152]., with the permission of AIP Publishing

In contrast, by using the same Monte Carlo model as shown in Figure 28, Li et al. have simulated the trapped charge distribution in SiO_2_ and found that the trapped charges could form several alternating positive and negative charge layers [152]. Figure 29 shows the trapped charge distribution at the primary electron energy of 5 keV, in which the charge layers exist merely along the beam incidence direction. The rigorous analysis shows that the first layer near the surface top is due mainly to the SEE, so it is positive; the sixth layer is due mainly to the trapping of primary electrons, so it is negative and has the deepest location. And the rest four middle layers are due mainly to charge drift, in which the deposited electrons and holes move to opposite directions by the establishing electric field, causing the separation of the charge layers. Furthermore, the authors have also simulated the trapped charge distributions at greater primary energies, whereas the distribution ranges of the charge layers, especially along the depth dimension, remained almost unchanged, since the involved charging effects were all negative in which the primary electrons were decelerated to a similar landing energy of about 2.5 keV in the simulation.

### 4.4. Minimization of charging effect

The fact that the charging effect plays mostly harmful roles in experiments highlights the necessity of developing measures to minimize it as much as possible. This section is to introduce some measures that have been experimentally proven workable for minimizing the charging effect, which with the knowledge imparted by previous sections are expected to be understood more easily.

The first measure is to coat the insulating sample with a grounded conducting film with a thickness of several nm. This can eliminate the electric field outside the sample completely and thus can prevent the primary electron beam from being deflected. However, there is still an inner electric field inside the coated sample, but its strength is reduced by the induced charges accumulated at the sample-film interface, which have the sign opposite to the trapped charges in the sample. However, sample coating can change the surface condition and, thus, affect the emission of desired signals from the sample. It is therefore necessary to take other measures to minimize the charging effect in the situation that the sample coating is not allowed. One such solution is to neutralize the trapped charges. For example, imaging an insulating sample using SEM at a high primary energy, such as 30 keV, is rather likely to charge it negatively. For this situation, the neutralization can be achieved by using an auxiliary electron gun mounted in the SEM chamber to inject another electron beam with an energy of 0.1–3 keV to the sample [19], which, in this low range of primary energy, can introduce net positive charges into the sample, giving rise to the neutralization of the negative charges. Following the neutralization spirit gases, such as, water vapor, N_2_, He, or Ar can be injected into the high-vacuum SEM chamber, converting the conventional SEM into the variable-pressure SEM (VP-SEM). Its principle is that the incident primary electrons and the emitted secondary electrons can ionize the gas, in which ions or electrons due to ionization can be controlled to move to the sample by the electric field artificially set in the chamber for neutralizing the accumulated charges in the sample. Based on this VP-SEM has produced fine image contrast for insulating materials [20]. This idea has also been realized in EBL, forming the variable-pressure EBL (VP-EBL) [153]. In particular, the charging effect in oxide characterizations using SEM [23] and AES [154–156] can be minimized after injecting O_2_ into the instrument chamber, but a different principle is involved. The charging effect in these cases is mainly related to the oxygen vacancies on the sample surface due to electron-stimulated desorption (ESD) and they can be effectively eliminated by the injected O_2_, thus minimizing the charging effect. Heating the sample is another workable measure via releasing the trapped charges and it has been verified to improve the SEM image quality [24]. If possible, it is suggested to perform the experiment at the second critical primary electron energy, which can eliminate the pattern distortion in EBL [29,157] and improve SEM imaging [25]. The second critical primary electron energy can be known via the SEM imaging shown in Figure 18 [28] or via measuring the surface potential at two primary electron energies [158]. Furthermore, SEM imaging can also be improved by either taking the scanning electron beam to be a pulsed one [26] or altering the scanning mode [21,22].

## 5. Conclusions

(1) The charging effect induced by electron beam irradiation can be categorized as either positive or negative charging effects.

Positive charging effect requires that the total electron yield at the beginning of the electron beam irradiation to be greater than the unity and surface potential is positive, and occurs for semi-infinite samples between the first and the second critical primary energies. In contrast, negative charging effect requires that the total electron yield at the beginning of the electron beam irradiation to be smaller than the unity and surface potential is negative, and occurs for semi-infinite samples either below the first critical primary energy or greater than the second critical primary energy. In particular, the second critical primary energy is important and can range 1–3 keV for most insulating materials.

(2)The positive surface potential impedes the emission of low-energy secondary electrons, reducing the total electron yield, via which the steady state of positive charging effect can be reached. The negative surface potential decelerates the primary electrons, improving the total electron yield, via which the steady state of negative charging effect can be reached.

Due to the low-energy nature of secondary electrons, the surface potential in the steady state of positive charging effect is small, in the order of several volts for semi-infinite samples. However, the surface potential in the steady state of negative charging effect can be great, which is linearly proportional to the primary energy, leading the landing energy to be approximately the second critical primary energy in the reduced total electron yield curve.

(3)Positive and negative charging effects can, respectively, reduce and improve the electron yield in electron yield measurement, make the secondary electron image intensity darker and brighter in SEM imaging, and shift the secondary electron peak in the emitted electron energy spectrum towards the lower and the higher energy sides.

In addition, since the surface potential in positive charging effect is usually on the order of several volts, it mainly impacts secondary electrons but not backscattered electrons. However, negative charging effect can be strong, leading the influence it exerts on experiments to be much more severe, such as the mirror imaging and the pattern displacement in EBL.

(4)Charging effect is not desired in most cases, highlighting the necessity of taking measures to minimize it. Measures such as coating the conducting film, neutralizing the accumulated charges by opposite charges or by gas atmospheres, setting the primary energy of electron beam at its second critical value, rising the temperature, etc., have been introduced. The knowledge on the charging effect imparted by the previous sections in this paper is expected to help people to better understand these measures.
